# Impaired membrane lipids in ischemic stroke: a key player in inflammation and thrombosis

**DOI:** 10.1186/s12974-025-03464-w

**Published:** 2025-05-28

**Authors:** Qian Wang, Dandan Wang, Yan Gao, Jie Jiang, Minghui Li, Shuhui Li, Xiaowen Hu, Jinfeng Wang, Tianqi Wang, Juan Zhang, Lei Feng, Chao Quan, Ping Zhang, Lan Zheng, Chunling Wan

**Affiliations:** 1 Bio-X Institutes, Key Laboratory for the Genetics of Developmental and Neuropsychiatric Disorders, Ministry of Education, Shanghai Jiao Tong University, 1954 Huashan Road, Shanghai, 200030 China; 2 Department of Neurology, Minhang Hospital, Fudan University, 170 Xinsong Road, Shanghai, 201100 China; 3 Instrumental Analysis Center, Shanghai Jiao Tong University, Shanghai, 200240 China; 4 Department of Neurology, Shanghai Medical College, Huashan Hospital, Fudan University, Shanghai, 200040 China; 5 Department of Hyperbaric Oxygen and Neurology, Naval Medical Center of PLA, Naval Medical University, Shanghai, 200052 China

**Keywords:** Ischemic stroke, Lipidome, Membrane lipids, Lipid signalling pathway, Inflammation, Thrombosis

## Abstract

**Background:**

Membrane lipids play a crucial role in brain function and cell signalling, and they serve as key biological substrates in inflammatory responses, thrombosis, and energy metabolism. Multiple clinical and molecular evidences suggest that membrane lipids are probably involved in the pathogenesis of ischemic stroke (IS). However, current knowledge about the membrane lipid landscape and its involvement in IS pathophysiology is limited.

**Methods:**

We performed untargeted lipidomic analysis on erythrocyte membranes from 56 IS patients and 55 healthy controls. Integrated with gene expression and weighted gene co-expression network analysis, we identified dysregulated lipid signalling pathways and their contributions to IS pathophysiology.

**Results:**

A total of 1392 erythrocyte membrane lipids were detected and quantified. Our results revealed significant impairment of membrane lipid homeostasis in IS patients, characterized by a marked reduction in glycerophospholipids (GPLs) and lysophospholipids (LPLs). Further analysis indicated that the impaired lipids were primarily concentrated in three disturbed signalling pathways, including the phospholipase A2-mediated GPL-LPL pathway, the phospholipase C-mediated inositol 1,4,5-trisphosphate/diglyceride pathway, and the sphingosine-1-phosphate (S1P)-S1P receptors pathway. Gene expression results indicated that these pathways were inhibited during the subacute phase of IS. Furthermore, these lipid signalling pathways form a highly interconnected network that collaboratively contributes to inflammation and thrombosis in IS, thereby influencing the progression and prognosis of the disease.

**Conclusion:**

Our findings reveal impaired erythrocyte membrane lipid homeostasis in IS, which implicates inflammatory processes and thrombosis in IS. This research offers new insights into the role of membrane lipids in IS pathogenesis, potentially informing future monitoring and therapeutic strategies.

**Supplementary Information:**

The online version contains supplementary material available at 10.1186/s12974-025-03464-w.

## Introduction

Stroke is the second leading cause of death worldwide and a leading cause of disability [1]. Ischemic stroke (IS) constitutes more than 80% of all stroke cases. The pathogenesis of stroke typically starts with the substantial accumulation of lipids and immune cells in subendothelial regions, called plaques. Plaque rupture induces platelet activation and aggregation at the site of injury, resulting in thrombus formation [2, 3]. Thrombosis can obstruct blood vessels, leading to severe neurological damage caused by the interruption of cerebral blood flow. Effective treatments for IS are limited to intravenous thrombolysis within 4.5 h and mechanical thrombectomy within 24 h [4]. However, only approximately 10% of patients can benefit from these therapies owing to the narrow treatment time window and the related risks of cerebral haemorrhage [5]. A deeper understanding of the pathological mechanisms of IS is urgently needed to identify new therapeutic targets and extend the treatment window, further improving IS management and prognosis.

Increasing evidences underscore that lipid metabolism disorders are deeply involved in the pathophysiology of IS. Lipids are major components of brain tissue and are crucial for maintaining the normal structure and function of the brain in ischemia-induced pathological environments [6]. Low-density lipoprotein cholesterol (LDL-C), triglycerides (TGs), and total cholesterol (TC) are possible risk factors and predictors of IS [7–9]. Additionally, some lipid molecules act as inflammatory mediators involved in regulating the inflammatory response, which is the core pathobiology of IS and contributes to its onset, progression, and outcome. For example, arachidonic acid (AA) metabolites regulate the occurrence and resolution of inflammation, platelet aggregation, and synaptic function [6]. Sphingosine-1-phosphate (S1P) signalling is involved in lymphocyte trafficking, inflammatory mediator synthesis, and endothelial function maintenance [10]. In addition, recent meta-analyses suggest that high omega-3 polyunsaturated fatty acid (PUFA) levels are associated with a lower risk of IS [11]. Overall, the available research highlights the crucial role of lipids in IS. However, the evidence is scattered, and the precise physiological mechanisms of lipids in IS remain unclear. Furthermore, some signalling lipids share common modifying enzymes and downstream targets, forming a highly interconnected lipid signalling network [12]. A systematic and in-depth exploration of lipid metabolism characteristics in IS will aid in uncovering the biochemical substrates involved in the pathological mechanisms of IS, driving the discovery of new therapeutic targets.

Lipidomics provides a powerful tool for comprehensively characterizing lipid profiles in tissue or fluid samples, thereby facilitating a deeper understanding of the lipid landscape in IS. Peripheral lipidomics has revealed increases in ceramides (Cers), TGs, and diglycerides (DGs) in IS patients, with decreases in phosphatidylcholine (PCs) and phosphatidylethanolamines (PEs) [13–18]. However, owing to objective methods such as mass spectrometry and lipid databases, early studies reported a limited number of lipid molecules and focused on a few lipid classes. In addition, complex lipids consist of a specific backbone architecture conjugated to fatty acids with different saturations and chain lengths, thus conferring unique physicochemical properties and physiological functions. However, some studies have focused primarily on the total content of lipid classes, with less emphasis on the changes in individual lipid molecules and specific acyl chains in IS. Furthermore, existing studies have focused mainly on differential lipids, with limited investigations into the regulation underlying lipid disturbances in the pathological state of IS. Finally, compared with body fluids such as blood or cerebrospinal fluid, the erythrocyte membrane has greater stability and reduced susceptibility to metabolic or pharmacological influences. Studies have shown that lipid status in the brain is related to the composition of erythrocyte membrane lipids [19–22]. However, there are no comprehensive studies on erythrocyte membrane lipidome in IS.

In this study, we systematically characterized the lipid profile of erythrocyte membranes in patients with IS via UHPLC‒MS/MS, which can be used to measure various lipid categories quantitatively and rigorously. We identified dysregulated lipid molecules and signalling pathways and further explored the role of impaired membrane lipid homeostasis in the pathological mechanisms of IS. This study provides new insights into the potential roles of membrane lipids in the pathogenesis and pathophysiology of IS.

## Methods

### Study participants

Patients with IS were recruited from the Department of Neurology of Minhang Hospital of Fudan University (Shanghai, China). The IS diagnosis was established through comprehensive brain magnetic resonance imaging (MRI) and computed tomography (CT), following the criteria outlined by the American Heart Association Stroke Committee. National Institutes of Health Stroke Scale (NIHSS) score was used to evaluate the disease severity. The exclusion criteria for IS patients included: (1) severe comorbidities, such as malignant tumors or severe organ dysfunction; (2) dementia, brain trauma, or other complex neuropsychiatric disorders; and (3) pregnancy. Healthy controls (HC) were recruited from the Physical Examination Center of Minhang Hospital of Fudan University. The inclusion criteria for HC were as follows: (1) no history of stroke; (2) no mental or neurological disorders; (3) no carotid artery and cerebrovascular disease and other organic illness; and (4) no allergic skin diseases, asthma, vasculitis, or other immune system disorders. The exclusion criteria for HC were: (1) binge drinking or excessive alcohol consumption in the last 24 h; (2) drug abuse or dependence; (3) taking drugs within two weeks; and (4) pregnancy. The clinical data of participants, including symptoms, laboratory results, clinical outcomes, and other examination findings at admission, were retrospectively collected from medical records. This study conformed to the principles outlines in the Declaration of Helsinki, and was approved by the Ethics Committee of Minhang Hospital of Fudan University (ID 2021-024-01 K). All subjects provided informed consent before participation.

### Sample collection and processing

Blood of participants in the IS and HC groups was collected after an overnight fasting. All blood samples were collected during the subacute phase of IS patients [23], with 47 samples collected 1–2 days post-stroke and 8 samples collected 3–4 days post-stroke. The plasma, leukocytes, and erythrocytes were isolated using centrifugation at 1,600 g for 10 min at 4 °C. The white blood cells were then washed with red blood cell (RBC) lysis buffer (Tiangen, Beijing, China) and subsequently 1 mL of TRIZOL (Invitrogen, Carlsbad, CA, USA) reagent was added to extract total RNA. The plasma, leukocytes, and erythrocytes were stored at -80 °C until further use.

### Clinical indicators detection

Peripheral immune cells were analyzed using the automated blood cell analyzer, including white blood cell count (WBC), neutrophils (Neu), lymphocytes (Lym) and monocytes (Mono). The maximum platelet aggregation rate (MPAR) was measured by light transmission aggregometry. Thromboelastogram (TEG) was analyzed using TEG coagulation analyzer. TEG contains five indices, including coagulation index (CI), maximum amplitude (MA), alpha angle (α), reaction time (R), and clotting time (K).

### RBC membrane lipid extraction

Erythrocytes were treated overnight in 10x tris-HCL to lyse them. After vortexing, the mixture was centrifuged at 16,000 rpm for 15 min, followed by washing the erythrocytes three times with phosphate-buffered saline to eliminate haemoglobin residues. Lipids were extracted from the erythrocyte membrane precipitates using chloroform/methanol mixture (2:1, v/v). 0.25 mg of CL (14:0) was added to each sample as an internal standard. The extraction process was performed at 40 °C for 2 h, and then dried by vacuum centrifuge. The dried powder was dissolved in the dichloromethane/isopropanol/methanol mixture (1:1:1, v/v/v), and the supernatant obtained after centrifugation was used for subsequent detection.

The main lipids were quantitatively analyzed by using 6 lipid classes of standard substances, including SM (d18:1/12:0), Cer (d18:1/18:1), PS (17:0/17:0), PE (17:0/17:0), PC (19:0/19:0), TG (17:0/17:1/17:0) D5. The standard substances were dissolved at 0.5 mg/mL in methanol/chloroform (1:1, v/v) and gradient diluted with dichloromethane/isopropanol/methanol (1:1:1, v/v/v) to obtain an external standard curve with 9 concentration gradients, as shown in supplementary Table 1. Additionally, to monitor the stability of the lipid detection, equal volumes of samples were mixed as the quality control (QC) sample, with 1 QC injected every 10 samples. To avoid cross-contamination,1 blank control was added every 20 samples.

### UHPLC-MS/MS conditions

An ultra-high-pressure liquid chromatography (UHPLC) system coupled with a Q Exactive plus Mass spectrometer (QE-MS) (Thermo Fisher Scientific, Waltham, USA) was used to analyze the membrane lipid samples. ACQUITY UPLC BEH C18 (100 × 2.1 mm, 1.7 μm, Waters) was used to do the chromatographic separation. The column temperature maintained was at 60 °C and the injection volume was 1 µL. The mobile phase A consisted of water/acetonitrile (60:40) with 10 mM ammonium formate and 0.1% formic acid, and mobile phase B consisted of isopropanol/acetonitrile (90:10) with 10 mM ammonium formate and 0.1% formic acid. The elution gradient was as follows: The gradient of mobile phase A decreased from 95 to 1% in 17 min, and remained at 1% A for 3 min. The target compounds were analyzed in full scan/data-dependent MS2 (Full MS/dd-MS2) mode. The collision energy is NEC 15,30, 45 to fragment the ions. Nitrogen (99.999%) was used as collision-induced dissociation gas. The dd-MS/MS resolution was 17,500 with an AGC target of 2.00E + 05 and a maximum injection time of 50 ms. Samples were analyzed in both positive and negative modes, and the spray voltage was set to 3.2 kV (positive mode) and 3.0 kV (negative mode), and the capillary temperature was held at 320 °C. The S-lens RF level was set to 50.

### Lipidomics data processing

Data was processed by Lipidsearch 4.2 software (Thermo Fisher Scientific, Waltham, USA) for peak picking, alignment, and normalization to produce peak areas for retention time (Rt) and m/z data pairs. Identified lipids were subjected to further filtration based on the following quality control criteria: (1) ‘Reject’ = 0; (2) Grade A or B; (3) Coefficient of Variation (CV) for peak areas in QC samples below 30%. Lipid quantification was conducted using class-specific external standard curves, and molar concentrations were calculated from the lipid peak areas. The single peak area was normalized by the total peak area of the corresponding sample, then processed by ComBat model to correct the batch difference of lipid extraction and the system error of instrument operation [24]. 

### Enzyme-linked immunosorbent assay (ELISA)

The levels of inositol 1,4,5-trisphosphate (IP3), S1P and three inflammatory factors, including interleukin-6 (IL-6), interleukin-1β (IL-1β) and interleukin-10 (IL-10), in plasma were assessed using ELISA kits (Elabscience, Wuhan, China; USCN, Wuhan, China; and Billerica, MA, USA) according to the manufacture manual.

### Real-time PCR assays

Total RNA (1 µg) from leukocyte cDNA samples was reverse transcribed using the SuperRT cDNA Synthesis Kit (TaKaRa, Kyoto, Japan). Gene expression levels were measured by real-time PCR with the SYBR Green Master Mix (TaKaRa, Kyoto, Japan). The primer sequences for the target gene are listed in supplementary Table 2. *ACTB* served as the reference housekeeping gene, and the relative expression levels of the target gene were calculated by 2^−∆∆Ct^ method.

### Weighted gene co-expression network analysis (WGCNA)

WGCNA analysis can identify modules of highly correlated lipids, assess the association between modules and traits of interest, and determine key lipids. In this study, the peak areas of all detected lipids in the IS group were analyzed with the WGCNA R package to identify modules and key lipids associated with clinical indicators of inflammation and thrombosis. The peak areas underwent LOG(X + 1) transformation and outliers were removed. According to the principle of a scale-free network, soft thresholds (power = 5, R^2^ = 0.90) were selected to construct a scale-free co-expression network, and the adjacency matrix was converted into a topological overlap matrix (TOM). Lipids with similar expression patterns were categorized into lipid modules using average linkage hierarchical clustering, with each module containing at least 30 lipids. Subsequently, Pearson correlation coefficients between characteristic lipids of the modules and clinical indicators were calculated. The four modules with the strongest relevance to indicators of inflammation and thrombosis serve as key modules for subsequent analysis.

### Statistics

Software R 4.3.2 was used to conduct statistical analysis. Group comparisons in demographic characteristics were conducted using Mann-Whitney *U* test for continuous variables and chi-square test for categorial variables. Principal Component Analysis (PCA) and Orthogonal Partial Least Squares-Discriminant Analysis (OPLS-DA) were conducted using the MetaboAnalyst platform [25]. Multiple linear regression was employed to examine the differences in lipid profiles and gene expression between the IS and the HC groups, with adjustments for gender, age, and Body Mass Index (BMI). For genes, *p*-values were further adjusted using the False Discovery Rate (FDR) method. *p*-value < 0.05 (or FDR < 0.05) was considered as statistically significant. Spearman’s correlation test was utilized for correlation analysis. Hypergeometric distribution was used for enrichment analysis.

## Results

### Impaired membrane lipids in ischemic stroke

**Fig. 1:**
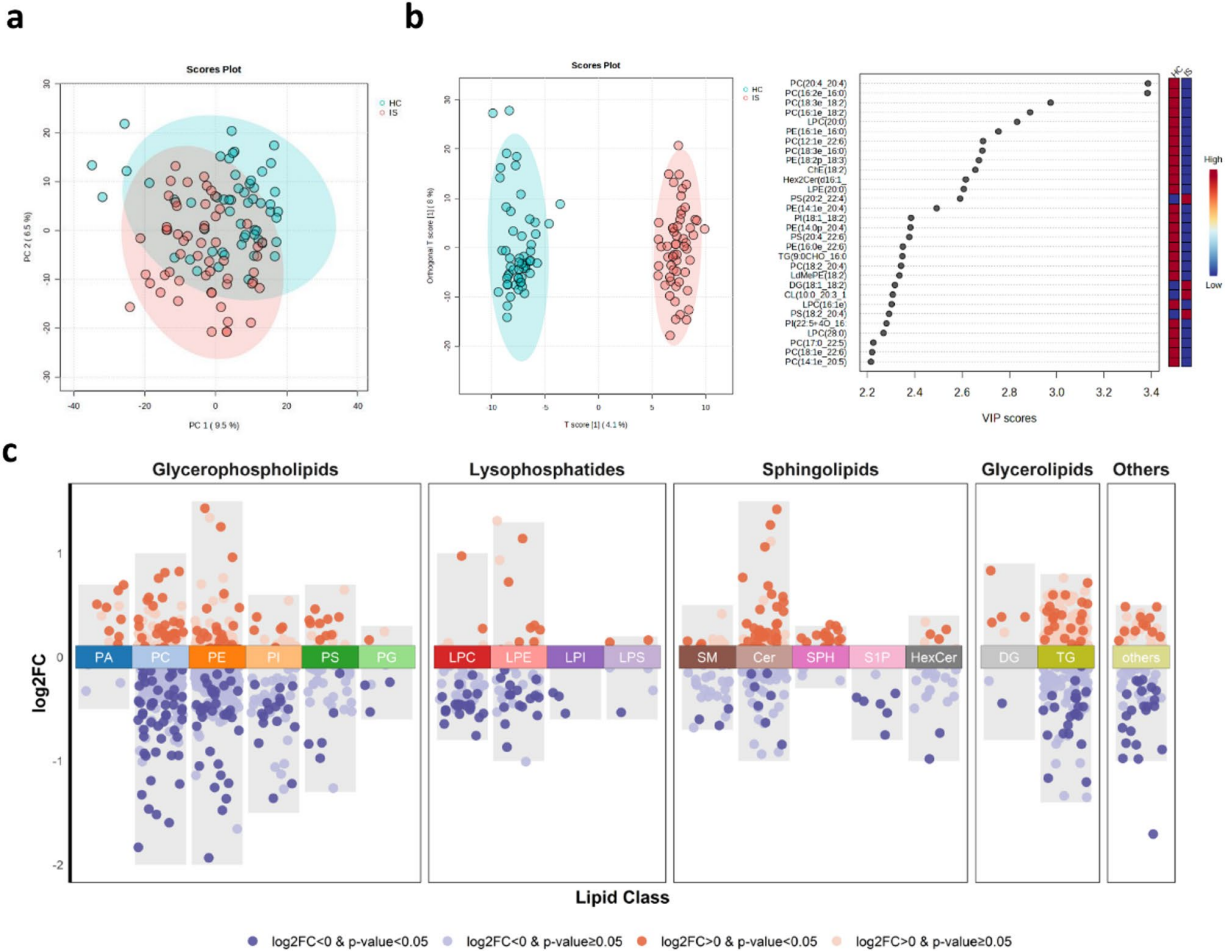
Impaired erythrocyte membrane lipid homeostasis in patients with ischemic stroke (IS). (**a**) 2D score plot of principal component analysis of IS and HC groups. (**b**) 2D score plot of orthogonal partial least squares discriminant analysis (OPLS-DA) of IS and HC groups (left); and the top 30 lipids with the highest variable importance in projection (VIP) scores given by OPLS-DA (right). (**c**) Differences in 1392 lipids between IS and HC groups. *p*-values were calculated by multiple linear regression adjusting for gender, age, and Body Mass Index. FC: Fold change; HC: Healthy controls

A total of 111 subjects were recruited for this study, including 56 IS patients and 55 HCs. The demographic and clinical characteristics are shown in Supplementary Table 3. An UHPLC-MS-based nontargeted lipidomic approach was used to profile 1392 lipids in 111 erythrocyte membrane samples (Supplementary Fig. 1, Supplementary Table 4). The lipids identified predominantly comprised glycerophospholipids (GPLs), lysophospholipids (LPLs), SPs and glycerolipids (GLs), which were further categorized into 17 lipid classes, including PC, PE, phosphatidylserines (PS), phosphatidylinositol (PI), phosphatidic acid (PA), phosphatidylglycerol (PG), lysophosphatidylcholine (LPC), lysophosphatidylethanolamine (LPE), lysophosphatidylserine (LPS), lysophosphatidylinositol (LPI), sphingomyelin (SM), Cer, hexosylceramide (HexCer), SPH, S1P, TG and DG. Among them, 1337 lipids were quantified in molar concentration (µM), enabling the statistical analysis of the total content of lipid classes or fatty acyl chain compositions. PCA of 1392 lipids revealed a distinct separation trend between the IS and HC groups (Fig. 1a). Furthermore, OPLS-DA revealed significant differences in erythrocyte membrane lipid profiles between the IS and HC groups (*R*^*2*^*Y* = 0.981, *Q*^*2*^ = 0.788, *p* < 0.01) (Fig. 1b).

Unsupervised cluster analysis revealed that IS patients presented a marked reduction in membrane lipid content (Supplementary Fig. 2); additionally, these significantly reduced lipids predominantly belonged to the GPL and LPL classes, including PC, PE, PI, LPC, LPE, and LPI (Fig. 1c). Specifically, the abundance of 226 lipids were significantly lower in IS patients than in HCs, with 118 being GPLs and 41 being LPLs, after adjusting for gender, age and BMI by multiple linear regression. In addition, according to the variable importance in projection (VIP) determined via OPLS-DA, 24 of the top 30 lipids that contributed to the differentiation between the IS and HC groups were GPLs and LPLs (Fig. 1b), and most of the GPLs (22/24) were decreased in the IS group, highlighting the decrease in GPLs and LPLs as key features of membrane lipid abnormalities in IS patients.

### Disturbed glycerophospholipid metabolism

**Fig. 2:**
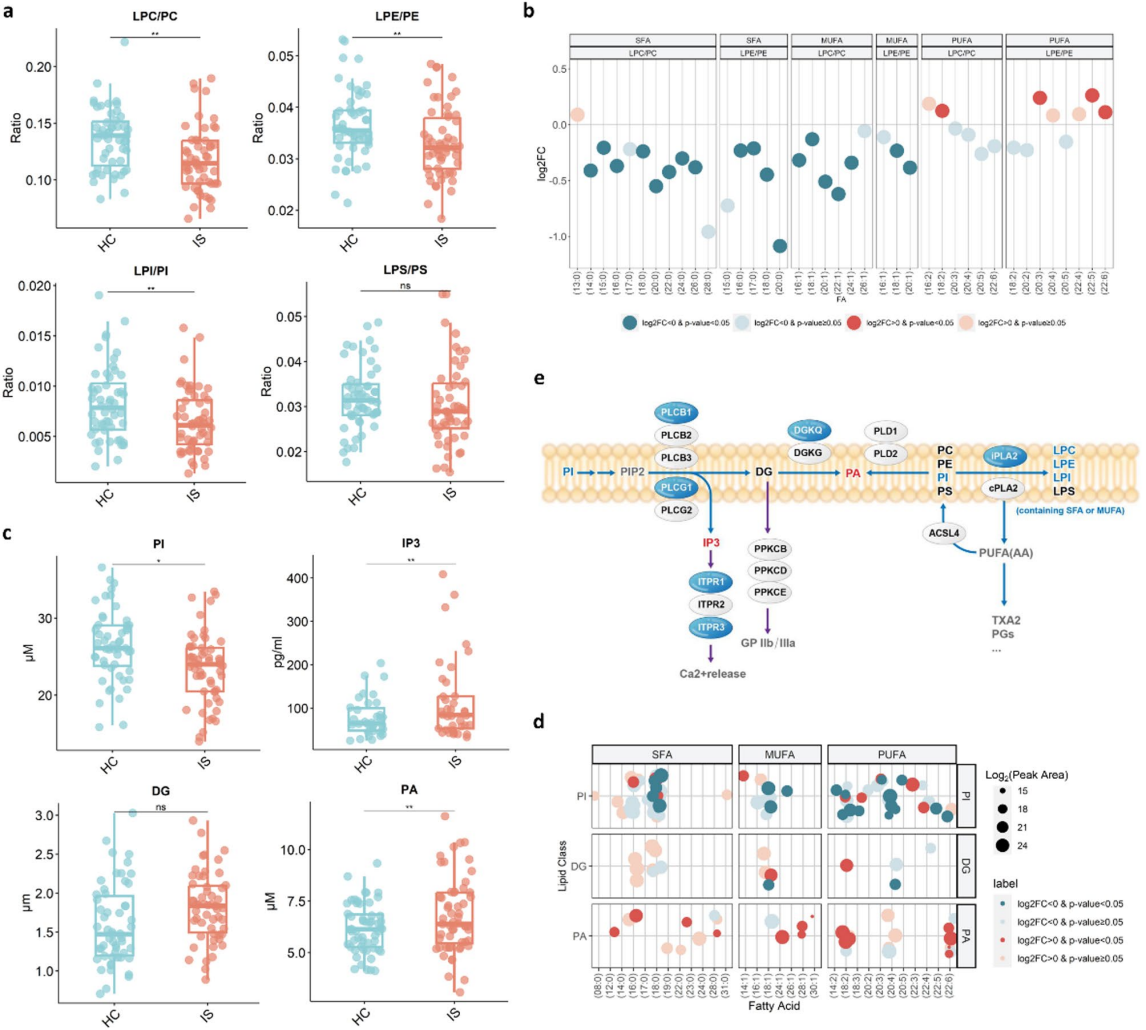
Disturbed glycerophospholipid (GPL) metabolism in patients with ischemic stroke (IS). (**a**) Ratios of LPLs to GPLs in patients with IS and healthy controls (HC), including LPC/PC, LPE/PE, LPI/PI, and LPS/PS. (**b**) Differences in the ratio of the total content of GPLs containing specific fatty acyl chains to the total content of LPLs containing specific fatty acyl chains between the HC and IS groups. (**c**) The total content of PI, IP3, DG, and PA in the HC and IS groups. (**d**) Differences of individual lipids of PI, PA and DG classes in patients with IS and HC. Each GPL contains two fatty acyl chains of different chain lengths and saturations. The horizontal coordinates indicate the individual fatty acyl chains contained in PIs, DGs, and PAs, so each lipid corresponds to two dots. Dot color represents significance and log_2_FC positive or negative, and dots size represents the log_2_(Peak Area). (**e**) Schematic diagram of changes in lipid contents and genes expression associated with GPL signalling pathways in the IS group compared to the HC group. Non-italics indicate lipids, and italics indicate genes. For lipids, blue letters indicate a significant decrease in the IS group, red letters indicate a significant increase in the IS group, and black letters represent no significant change. For genes, blue ovals represent significantly downregulated, and white ovals represent no significant change. Blue lines represent lipid synthesis or hydrolysis, and the purple lines represent regulatory targets or receptors. All *p*-values were calculated by multiple linear regression adjusting for gender, age, and Body Mass Index. *p < 0.05, **p < 0.01, ***p < 0.001. LPL: lysophosphatide; FC: Fold change; SFA: Saturated fatty acids; MUFA: Monounsaturated fatty acids; PUFA: Polyunsaturated fatty acids

On the basis of the observed decreases in individual GPLs and LPLs in the IS group, the total contents of LPC, LPE, and LPI were significantly lower (Supplementary Fig. 3), and the ratios of LPL/GPL in the IS group were significantly lower than those in the HC group (Fig. 2a), including LPC/PC (FC = 0.828, *p* = 0.004), LPE/PE (FC = 0.902, *p* = 0.004), and LPI/PI (FC = 0.755, *p* = 0.002). When focusing on the LPL/GPL ratios containing specific fatty acyl chains, such as LPC/PC-(16:0), i.e., the ratio of the content of LPC containing (16:0) to the content of PC containing (16:0), the results revealed that the ratios of LPL/GPL containing saturated fatty acids (SFAs) or monounsaturated fatty acids (MUFAs) generally decreased, whereas no similar trend was observed for PUFAs (Fig. 2b). GPLs usually contain an SFA or MUFA at the sn-1 position and a PUFA at the sn-2 position. Phospholipase A2 (PLA2) cleaves an ester bond at the sn-2 position of GPL, producing a free PUFA and an LPL containing SFA or MUFA, while acyl-CoA synthetase long-chain family member 4 (ACSL4) integrates PUFAs into membrane phospholipids. The expression levels of PLA2 (*PLA2G6* and *PLA2G4A*) and ACSL4 (*ACSL4*) were measured to investigate whether the reduction in LPL/GPL ratios containing SFAs or MUFAs was associated with abnormal PLA2 and ACSL4 activities. The results revealed a significant downregulation of *PLA2G6* expression, whereas *PLA2G4A* and *ACSL4* remained unchanged (Table 1; Fig. 2e). These findings indicate that downregulated PLA2 expression may slow the hydrolysis of PUFAs at the sn-2 position of membrane GPLs, leading to the reduced production of LPL-SFA/MUFA.

**Table 1:** The expression levels of genes related to disturbed lipid signalling pathways in ischemic stroke

Genes	Protein name	Ischemic atroke (mean ± SD)	Healthy control (mean ± SD)	Fold change	*p*-value^A^	FDR^B^
*PLA2G4A*	Cytosolic Phospholipase A2	0.976 ± 0.323	1.124 ± 0.454	0.868	5.85E-01	8.61E-01
***PLA2G6***	Calcium-Independent Phospholipase A2	0.732 ± 0.188	0.982 ± 0.222	0.746	1.00E-03	6.24E-03
*ACSL4*	Long-Chain Fatty-Acid-Coenzyme A Ligase 4	0.897 ± 0.24	1.014 ± 0.243	0.885	2.49E-01	4.48E-01
***PLCB1***	Phospholipase C Beta 1	0.799 ± 0.253	1.026 ± 0.277	0.779	4.66E-03	2.10E-02
*PLCB2*	Phospholipase C Beta 2	0.983 ± 0.271	1.033 ± 0.267	0.952	3.01E-01	4.93E-01
*PLCB3*	Phospholipase C Beta 3	1.034 ± 0.366	1.051 ± 0.446	0.984	1.76E-01	4.40E-01
***PLCG1***	Phospholipase C Gamma 1	0.691 ± 0.272	1.062 ± 0.431	0.650	1.04E-03	6.24E-03
*PLCG2*	Phospholipase C Gamma 2	0.93 ± 0.287	1.031 ± 0.29	0.902	6.44E-01	8.61E-01
***ITPR1***	Inositol 1,4,5-Trisphosphate Receptor Type 1	0.769 ± 0.29	1.048 ± 0.312	0.734	6.28E-03	2.25E-02
*ITPR2*	Inositol 1,4,5-Trisphosphate Receptor Type 2	0.904 ± 0.244	1.034 ± 0.257	0.874	2.00E-01	4.40E-01
***ITPR3***	Inositol 1,4,5-Trisphosphate Receptor Type 3	0.675 ± 0.261	1.019 ± 0.335	0.662	4.88E-04	6.24E-03
*PPKCB*	Protein Kinase C Beta	0.931 ± 0.32	1.066 ± 0.429	0.874	7.57E-01	8.79E-01
*PPKCD*	Protein Kinase C Delta	0.998 ± 0.318	1.026 ± 0.287	0.973	8.79E-01	8.79E-01
*PPKCE*	Protein Kinase C Epsilon	0.854 ± 0.369	1.007 ± 0.429	0.848	6.70E-01	8.61E-01
*PLD1*	Phospholipase D1	1.176 ± 0.319	1.035 ± 0.266	1.136	8.44E-01	8.79E-01
*PLD2*	Phospholipase D2	0.963 ± 0.233	0.988 ± 0.206	0.975	8.05E-01	8.79E-01
***DGKQ***	Diacylglycerol Kinase Theta	0.693 ± 0.331	1.024 ± 0.316	0.677	7.49E-03	2.25E-02
*DGKG*	Diacylglycerol Kinase Gamma	1.216 ± 0.395	1.072 ± 0.391	1.134	2.20E-01	4.40E-01
*ASAH1*	N-Acylsphingosine Amidohydrolase 1	0.948 ± 0.243	1.058 ± 0.471	0.896	9.50E-01	9.50E-01
*ACER3*	Alkaline Ceramidase 3	1.072 ± 0.357	1.069 ± 0.386	1.003	4.36E-01	6.54E-01
*SPHK1*	Sphingosine Kinase 1	0.887 ± 0.322	1.055 ± 0.434	0.841	9.47E-01	9.50E-01
***SPHK2***	Sphingosine Kinase 2	0.752 ± 0.239	0.99 ± 0.225	0.760	2.08E-02	4.68E-02
***S1PR1***	Sphingosine-1-Phosphate Receptor 1	0.504 ± 0.199	1.018 ± 0.364	0.495	7.94E-06	3.57E-05
*S1PR4*	Sphingosine-1-Phosphate Receptor 4	0.869 ± 0.27	0.995 ± 0.282	0.873	9.35E-01	9.50E-01
***S1PR5***	Sphingosine-1-Phosphate Receptor 5	0.388 ± 0.201	1.045 ± 0.568	0.371	7.82E-06	3.57E-05
***CERS2***	Ceramide Synthase 2	0.659 ± 0.178	0.976 ± 0.319	0.675	1.25E-03	3.75E-03
*CERS4*	Ceramide Synthase 4	0.908 ± 0.348	1.022 ± 0.265	0.889	2.93E-01	5.27E-01

^A^*p*-values were calculated by multiple linear regression adjusting for gender, age, and BMI

^B^ FDRs were adjusted by False Discovery Rate Correction

Among the 6 GPL classes identified, the decrease in PI was remarkable (Fig. 1c). Specifically, 77% (17/22) of PIs with significant differences were reduced in the IS group, and the total PI content was significantly lower in the IS group than in the HC group (FC = 0.919, *p* = 0.040; Fig. 2c and d). PI is phosphorylated to produce various phosphoinositides (PPIs), among which phosphatidylinositol-4,5-phosphate (PIP2) is central to this pathway [26]. PIP2 is hydrolysed by phospholipase C (PLC) into inositol 1,4,5-trisphosphate (IP3) and DG. DG and IP3 trigger the activation of protein kinase C (PKC) and Ca^2+^ release from internal stores, respectively. Although PIP2 was not detected in our dataset, the plasma IP3 levels (FC = 1.293, *p* = 0.008; Fig. 2c) were significantly increased in the IS group, and DG content had a significantly increased trend (FC = 1.248, *p* = 0.137; Fig. 2c). Diacylglycerol kinases (DGKs) phosphorylate DG to produce PA to regulate PLC activity. Our results revealed that all eight PAs with significant changes were increased, and that the PA content increased in the IS group (FC = 1.043, *p* = 0.009; Fig. 2c and d). Our findings suggest a potential disturbance in the PI-IP3/DG-PA pathway. We subsequently examined the mRNA levels of 15 genes involved in this pathway (Table 1; Fig. 2e), including the PLC, IP3 receptor (IP3R), PKC, DGK, and phospholipase D (PLD). The expression levels of 5 genes, namely, *PLCB1*, *PLCG1*, *ITPR1*, *ITPR3* and *DGKQ*, were significantly reduced in IS patients, whereas the expression of the remaining genes did not significantly change. The downregulation of *PLCB1* and *PLCG1* expression suggested the PI-IP3/DG pathway and its associated physiological functions were suppressed, and the accumulation of IP3 might result from the downregulation of IP3R (*ITPR1* and *ITPR3*) expression.

### Disturbed sphingolipid metabolism

**Fig. 3:**
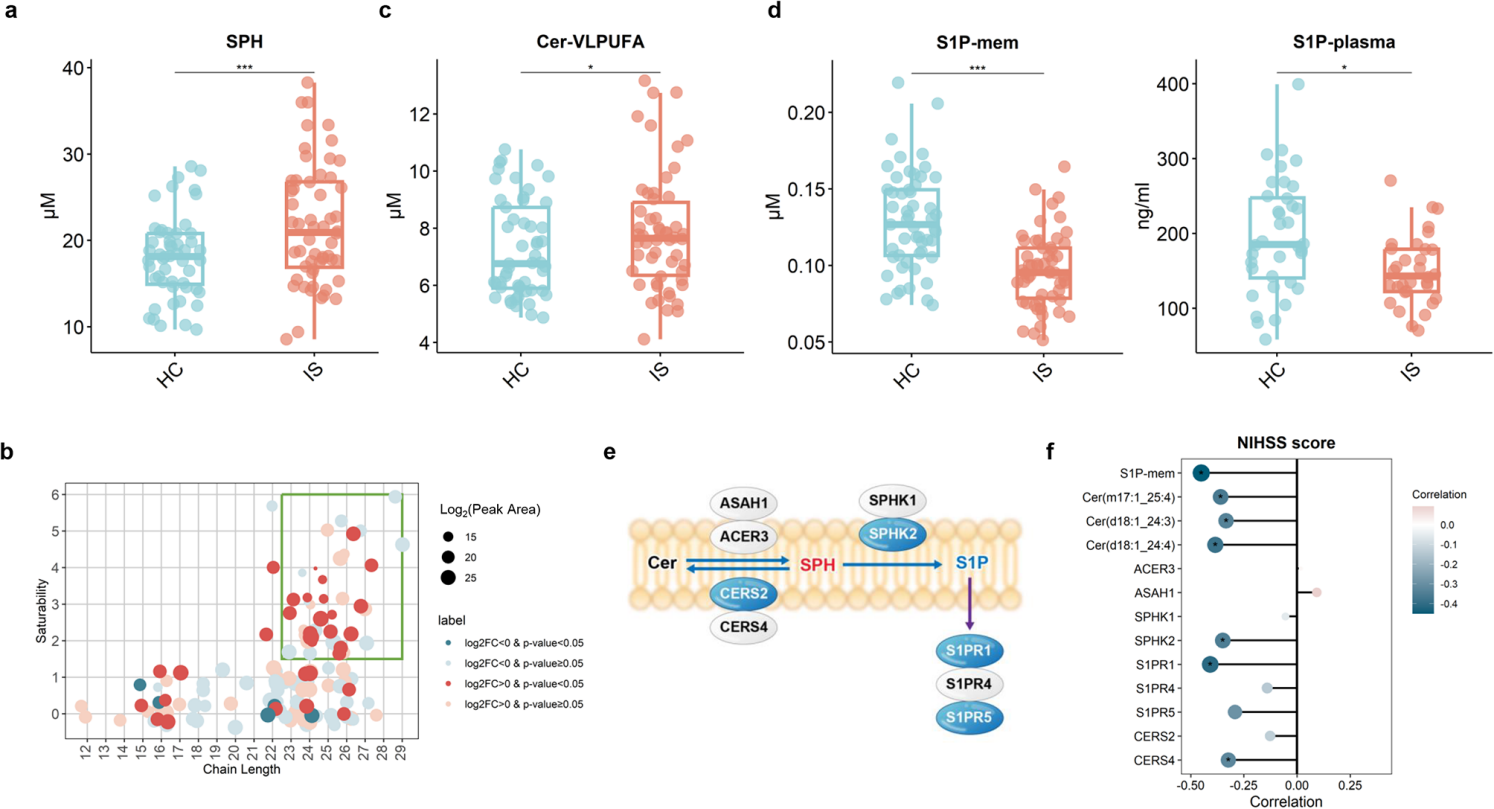
Disturbed sphingolipid (SP) metabolism in patients with ischemic stroke (IS). (**a**) The total content of sphingosine (SPH) in the healthy controls (HC) and IS groups. (**b**) Differences of individual lipids in ceramide (Cer) class between HC and IS groups. Each dot represents an individual lipid. Cer comprises a SPH backbone (usually chain length C18) attached to a variable chain length fatty acid (FA) via amide bon, and the horizontal and vertical coordinates indicate the chain length and saturability of the fatty acyl chains, respectively. Dot color represents significance and log_2_FC positive or negative, and dots size represents the log_2_ (Peak Area). Dots in the green box are Cer-VLPUFAs. (**c**) The total content of Cer-VLPUFA in the HC and IS groups. (**d**) The total content of erythrocyte membrane sphingosine-1-phosphate (S1P) (left) and plasma S1P (right) in the HC and IS groups. (**e**) Schematic diagram of changes in lipid contents and genes expression related to sphingolipid metabolism in the IS group compared to the HC group. Non-italics indicate lipids, and italics indicate genes. For lipids, blue letters indicate a significant decrease in the IS group, red letters indicate a significant increase in the IS group, and black letters represent no significant change. For genes, blue ovals represent significantly downregulated, and white ovals represent no significant change. Blue lines represent lipid synthesis or hydrolysis, and the purple lines represent regulatory targets or receptors. (**f**) NIHSS score was significantly correlated with sphingolipids and related genes in IS. *p*-values were calculated by multiple linear regression adjusting for gender, age, and Body Mass Index (**a**-**d**) or Spearman rank correlation Test (**f**) as appropriate. *p < 0.05, ***p < 0.001. FC: Fold change; VLPUFA: Very long chain polyunsaturated fatty acids; NIHSS: National institutes of health stroke scale

In contrast to the general decrease in GPLs and LPLs, the abundance of SPs showed an opposite trend in the IS group (Fig. 1c). The abundance of 59 SPs significantly increased in IS, whereas that of only 20 SPs decreased. The increased SPs comprised 35 Cers and 17 SPHs, with the total content of SPHs significantly increasing (FC = 1.153, *p* < 0.001, Fig. 3a). Notably, the increased Cers in IS group primarily contained very long-chain PUFA (VLPUFA) (Fig. 3b), and the total content of Cer containing VLPUFA (Cer-VLPUFA) increased significantly (FC = 1.132, *p* = 0.031, Fig. 3c). S1P showed the opposite performance to other sphingolipids. Although only 7 S1Ps on the erythrocyte membrane were identified, 6 of them were significantly decreased in the IS group (Fig. 1c), and the total S1P content was markedly reduced (FC = 0.753, *p* < 0.001, Fig. 3d). Given the low content of S1P on the cell membrane, we subsequently measured plasma S1P levels using ELISA, which also revealed a significant decrease in IS patients (FC = 0.773 *p* = 0.032, Fig. 3d).

The dysregulation of Cer, SPH, and S1P in IS prompted us to explore the Cer-SPH-S1P pathway, as these SPs can interconvert. Specifically, Cer is hydrolysed by ceramidases (CDases) to produce SPH, which can be recycled to Cer by ceramide synthases (CerSs) or phosphorylated by sphingosine kinases (SPHKs) to form S1P. S1P exerts its effects through five S1PR receptors (S1PR1-5). Therefore, we examined the expression levels of 9 genes involved in the Cer-SPH-S1P pathway, including CDase, CerS, SPHK, and S1PR. The results revealed a significant decrease in *SPHK2*, *S1PR1*, *S1PR5*, and *CERS2* in the IS group, whereas the remaining 5 genes were not significantly different (Table 1; Fig. 3e). On the basis of the sphingolipid profile and gene expression results, the accumulation of SPH and the reduction in S1P may result from the downregulation of *CERS2* and *SPHK2* expression, as this leads to reduced SPH consumption and diminished S1P production. Notably, the reduced S1P levels and downregulated *S1PR1* and *S1PR5* expression impaired S1P-S1PR signalling.

Furthermore, multiple sphingolipids and related enzymes exhibited significant inverse correlations with NIHSS scores, including three Cer-VLPUFAs (Cer(d18:1_24:3), Cer(d18:1_24:4), and Cer(m17:1_25:4)), erythrocyte membrane S1P, *CERS4*, *S1PR1*, and *S1PR5 *(Fig. 3f). These findings suggest that sphingolipids, particularly the S1P-S1PRs signalling pathway, may serve as indicators of disease severity and progression, highlighting their significant prognostic value in IS.

### Disturbed membrane lipid signalling pathways are synergistically involved in thrombosis and inflammation in patients with IS

**Fig. 4:**
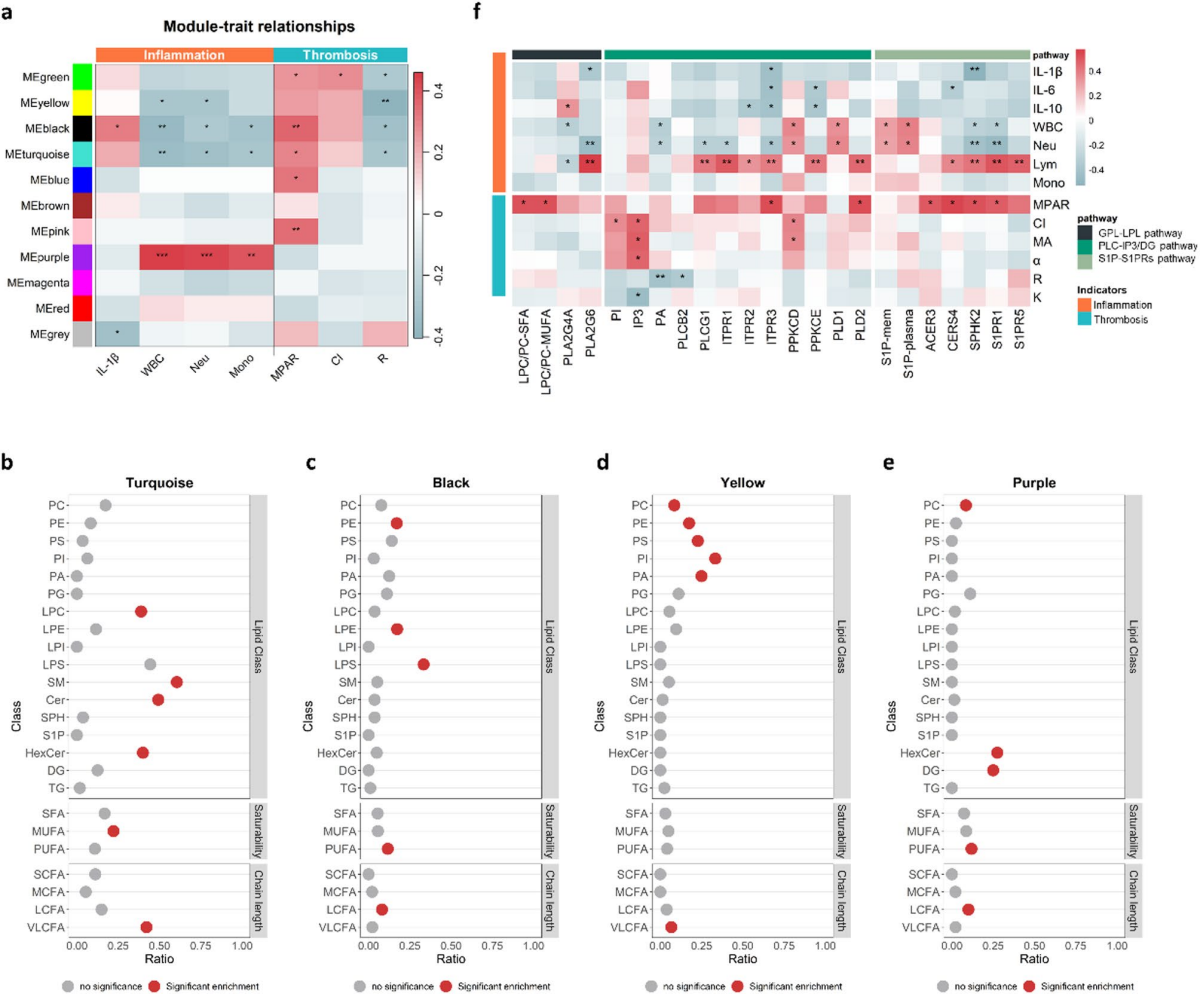
Disturbed lipid metabolism pathways are synergistically involved in inflammation and thrombosis in IS. (**a**) 11 modules revealed by the WGCNA. (**b**-**e**) The results of lipid enrichment analysis for the turquoise (**b**), black (**c**), yellow (**d**), and purple (**e**) modules. The ratio represents the proportion of a specific lipid classification within the module relative to the total lipid quantity. *p*-values were calculated by hypergeometric test, with *p* < 0.05 regarded as significantly enriched. (**f**) Spearman Rank correlation analysis of clinical markers of inflammation and thrombosis with genes and lipids in lipid signalling pathways in IS. *p < 0.05, **p < 0.01. IS: Ischemic stroke; NIHSS: National institutes of health stroke scale; WGCNA, Weighted gene co-expression network analysis; SFA: Saturated fatty acids; MUFA: Monounsaturated fatty acids; PUFA: Polyunsaturated fatty acids; SCFA: Short chain fatty acids; MCFA: Medium chain fatty acids; LCFA: Long chain fatty acids; VLCFA: Very long chain fatty acids; IL-6: Interleukin-6; IL-1β: Interleukin-1β; IL-10: Interleukin-10; WBC: White blood cell count; Neu: Neutrophils; Lym: Lymphocytes; Mono: Monocytes; MPAR: The maximum platelet aggregation rate; The five indices in the thromboelastogram, including CI, MA, α, R, and K. CI: Coagulation index, represents a composite score that reflects the overall coagulation status of the patient; MA: Maximum amplitude, measures the maximum strength of the clot; α: Alpha angle, represents the rate of clot formation; R: Reaction time, is the time taken for the initial clot formation to begin; K: Clotting time, measures the time taken for the clot to reach a certain amplitude (typically 20 mm)

In both the HC and IS groups, the genes involved in the impaired membrane lipids in IS exhibited significant positive correlations with one another (Supplementary Fig. 4). These findings suggest that these disrupted lipid signalling pathways may act synergistically in IS pathophysiology. To investigate the relationships between membrane lipids and pathophysiology in IS, we grouped lipids into 11 modules by applying WGCNA based on the similarity of lipid profiles, and then associated these 11 modules with indicators of inflammation and thrombosis (Fig. 4a). The inflammatory markers were assessed in both groups (Supplementary Fig. 5). WBC, Neu, Mono, and IL-6 in IS patients were significantly higher compared to those in HCs. IL-10 displayed an upward trend. Lym showed a downward trend, while no notable changes were detected in IL-1β.

The results revealed that 3 modules, namely, the yellow, black and turquoise modules, were significantly correlated with clinical indicators of both inflammation and thrombosis. These 3 modules were significantly negatively correlated with WBC, Mono, Neu and R, and significantly positively correlated with MPAR (Fig. 4a). These results indicate that the lipids in these 3 modules may play dual roles in inflammation and thrombosis. Additionally, the purple module displayed strongly positive correlations with inflammatory indicators, including WBC, Neu, and Mono, underscoring the pivotal role of the lipids in this module in the inflammatory response. Given the strong correlation between these 4 modules and indicators of inflammation and thrombosis, they were regarded as key modules of interest. Enrichment analysis revealed that lipids significantly enriched in these 4 modules highly overlapped with dysregulated membrane lipids in IS (Fig. 4b–e). Specifically, LPLs were enriched mainly in the turquoise module (LPCs) and black module (LPEs and LPSs). PUFA-containing GPLs, including PI and PA were enriched in the yellow module, and DGs were enriched in the purple module. Cers were enriched mainly in the turquoise module, and 67.16% of the Cers in this module contained very long-chain fatty acids. Additionally, S1Ps were enriched in the blue module (Supplementary Fig. 6), which was significantly positively correlated with MAPR.

On the basis of the WGCNA results, Spearman correlation analysis was used to determine the direct correlations of key lipids and genes with clinical indicators of thrombosis and inflammation (Fig. 4f). Interestingly, multiple lipids and genes were significantly correlated with indicators of thrombosis and inflammation in IS patients. For example, Lym was significantly positively correlated with *PLA2G6*, *PLCG1*, *ITPR1*, *ITPR2*, *ITPR3*, *PPKCE*, *PLD2*, *CERS4*, *SPHK2*, *S1PR1*, and *S1PR5*. MPAR was significantly positively correlated with LPC/PC-SFA, LPC/PC-MUFA, *ITPR3*, *PLD2*, *ACER3*, *CERS4*, and *S1PR1*. In addition, CI was significantly positively correlated with PI, IP3, and *PPKCD*. The S1P levels in plasma and on the erythrocytic membrane were significantly positively correlated with Lym and WBC. Taken together, these results demonstrate that the disturbance of membrane lipid signalling pathways synergistically participates in inflammation and thrombosis in IS, thereby affecting disease progression.

## Discussion

This study revealed impaired erythrocyte membrane lipid homeostasis in IS using non-targeted lipidome, characterized by reduced GPLs and LPLs. The imbalance is linked to disruptions in three key lipid signalling pathways: GPL-LPL, IP3/DG, and S1P-S1PRs pathways, which was validated by altered gene expression regulating these pathways. Furthermore, these pathways form an interconnected network, driving thrombosis and inflammation in IS, ultimately influencing disease progression and prognosis.

### Pathological implications of impaired membrane lipids in IS

RBC plays a critical role in IS by maintaining oxygen transport, microcirculatory homeostasis, and antioxidant capacity. The disturbances of erythrocytic membrane lipid significantly impacts their structure and function in IS patients. Specifically, reduction levels of GPLs (PC, PE, and PI) impair membrane fluidity and stability, diminishing RBC deformability and compromising microcirculation. The decrease in PI and LPL disrupts ion channel activity, calcium homeostasis and signal transduction, while the increase in pro-apoptotic Cer and SPH potentially induces RBC apoptosis, exacerbating oxygen supply deficits in ischemic regions [27–29]. Notably, these alterations have systemic implications, with RBC membrane lipid alterations reflecting disturbances in brain lipid homeostasis [21, 22, 30–33], which impair neurotransmitter signalling and blood-brain barrier function, aggravating neuronal injury [27, 34–36]. 

Thrombosis and inflammation are central to IS pathology. The extensive correlations between key lipids, genes, and markers of inflammation and thrombosis highlight the critical role of membrane lipid imbalance and signalling dysregulation in these processes. PLA2 hydrolyses membrane GPLs to release free AA and LPC, collectively driving thrombosis. LPC promotes platelet aggregation and plaque formation [37, 38]. TXA2, an AA metabolite, is a classical platelet agonist that activates PLC to generate IP3 and DG, the core processes of thrombosis [39]. The binding of IP3 to IP3Rs initiates platelet activation and the release of agonists, creating a positive feedback loop [39]. DG activates PKC, promoting platelet adherence. These align with the positive correlations among IP3, *ITPR3*, and *PPKCD* with pro-thrombotic markers. Furthermore, the role of S1P in thrombosis remains controversial. Our study provides supportive evidence that *S1PR1* and *SPHK2* are significantly positively correlated with the MPAR and PLC-IP3/DG pathway-related genes in IS. This suggests that S1P/S1PR1 signalling may indirectly influence platelet aggregation by modulating the PLC-IP3/DG pathway, thus affecting platelet responses to classical agonists [40–43]. 

For inflammatory process, LPLs and AA metabolites act as critical inflammatory mediators, regulating the initiation, progression, and resolution of inflammation in IS [44–48]. The PLC-IP3/DG pathway modulates immune responses through Ca2 + release and PKC activation [49], while the S1P-S1PR1 signalling mediates lymphocyte migration and cytokine production [10]. The strong correlations among genes support the synergistic interactions between these lipid pathways. S1P regulates PLC signalling [50], and S1P and IP3 collectively elevate intracellular Ca2 + levels [51], activating PLA2 and accelerating the breakdown of membrane GPLs to free AA and LPLs. Furthermore, this extensive interplay between thrombosis and inflammation supports the concept of thromboinflammation, which drives stroke, myocardial infarction, and venous thromboembolism [52]. Our evidence supports the role of disturbed membrane lipid pathways in the partnership of inflammation and thrombosis in IS.

### Compensatory suppression of dysregulated lipid pathways in the subacute phase of IS

**Fig. 5:**
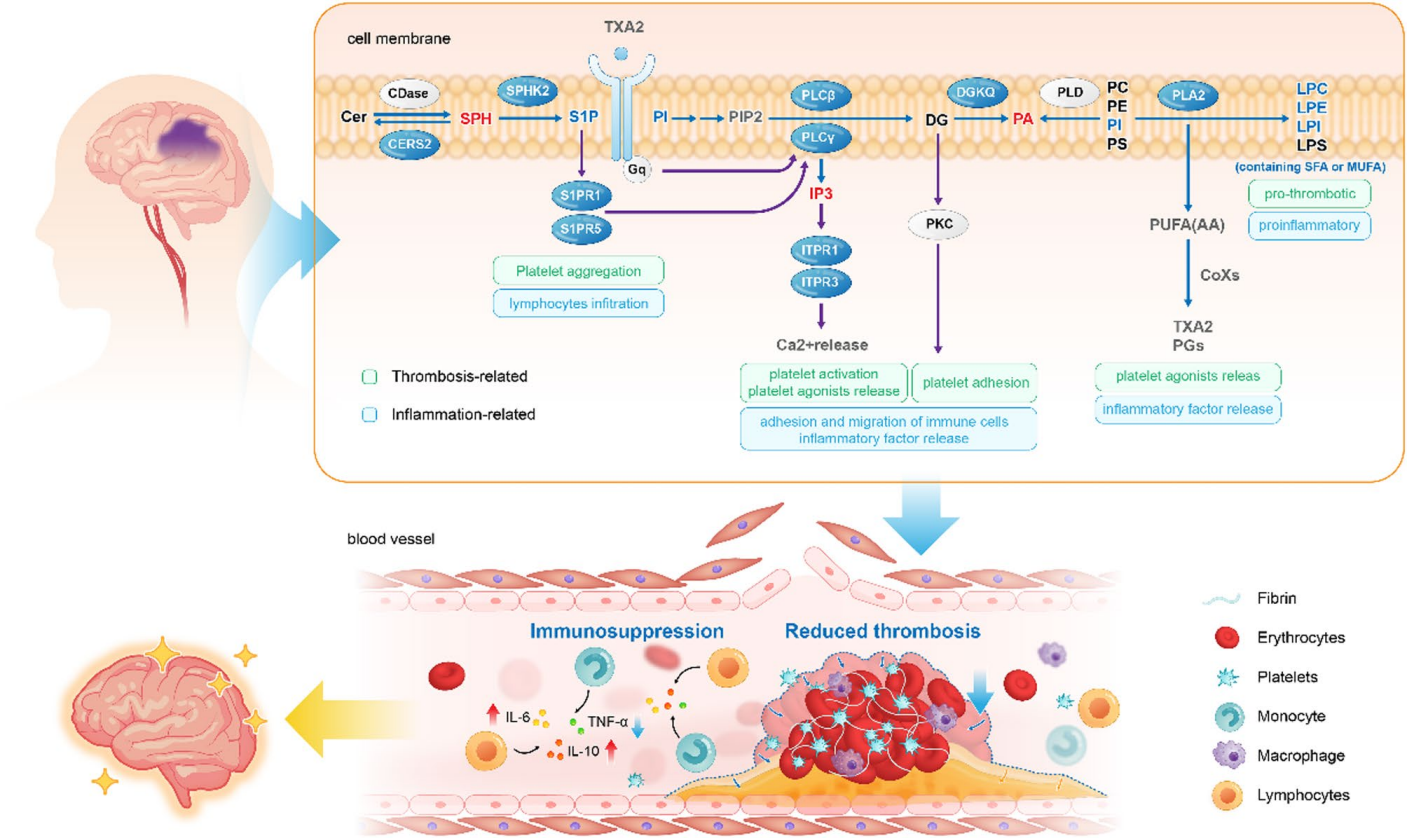
Pathological mechanisms associated with impaired membrane lipid homeostasis in ischemic stroke (IS). Membrane lipid homeostasis is impaired in patients with IS. The impaired lipids are primarily concentrated in three disturbed lipid signalling pathways: GPL-LPL, IP3/DG, and S1P-S1PRs pathway. These lipid pathways interact to form a highly interconnected lipid signalling network that collectively participates in thrombosis and the inflammatory responses of IS. However, during the subacute phase following IS, the key gene expressions and lipid levels significantly decrease, including a decline in the expression of PLA2, PLCs, IP3Rs, SPHK2, and S1PRs, as well as a reduction in the levels of PI, LPLs containing SFA or MUFA, and S1P. This indicates that these pathways are suppressed during this phase, thereby diminishing their roles in mediating inflammatory responses and thrombosis, which ultimately reduces pathological damage and accelerates homeostasis restoration. PLA2: Phospholipase A2; GPL: Glycerophospholipid; LPL: Lysophospholipid; PLC: Phospholipase C; IP3: Inositol 1,4,5-trisphosphate; DG: Diglyceride; SPHK2: Sphingosine kinase 2; S1P: Sphingosine-1-phosphate; S1PR: S1P receptor; IP3R: IP3 receptor; PI: Phosphatidylinositol; SFA: Saturated fatty acids; MUFA: Monounsaturated fatty acids

Interestingly, the overall downregulation of gene expression indicates the compensatory inhibition of these lipid signalling pathways in the subacute phase of IS, potentially as a protective mechanism. The study mainly analyzed IS patients 1 to 2 days post-stroke, corresponding to the subacute phase, when the body aims to mitigate pathological damage and promote recovery [53]. Downregulated PLA2 expression (*PLA2G6*) reduced the production of LPC and AA metabolites, thereby diminishing their proinflammatory and prothrombotic effects. Notably, the fatty acyl composition of LPCs is crucial for their physiological functions. LPC-SFA/MUFA is proinflammatory and proatherosclerotic, whereas LPC-PUFA appears to have the opposite effect [54, 55]. In IS patients, LPC-SFA/MUFA levels significantly decreased, while LPC-PUFA remained unchanged, indicating an attenuation of the harmful effects mediated by LPCs. Concurrently, reduced expression of PLCs (*PLCB1* and *PLCG1*) and IP3Rs (*ITPR1* and *ITPR3*) suppressed the PLC-IP3/DG pathway, reducing platelet activation and NF-κB-mediated inflammation [56, 57]. Prior studies reported that IP3R expression was strongly decreased at 4 h and undetectable at 16 h [58, 59], and IP3R-deficient cells exhibit increased resistance to toxic stimuli [60], further supporting the notion that its downregulation serves as a protective mechanism. Additionally, decreased S1P levels and downregulated *S1PR1* and *S1PR5* expression reduce lymphocyte infiltration and microvascular thrombus formation [61]. These findings underscore the critical role of membrane lipids in driving compensatory strategies during the subacute phase of IS, where they mitigate inflammation and thrombosis to facilitate tissue repair (Fig. 5).

### **Clinical and prognostic implications of impaired membrane lipid in IS**

This study highlights that membrane lipid dysregulation likely precedes IS and plays a dual role in its pathogenesis and progression [9, 62, 63]. For instance, hypertension and diabetes, as risk factors for IS [6, 64], are linked to lipid metabolic disturbances, including membrane lipids [65–69]. Impaired membrane lipids contribute to IS by regulating erythrocyte function, inflammation, and thrombosis. Thus, targeting key membrane lipids in the GPL-LPL, PLC-IP3/DG, and S1P-S1PRs pathways may aid IS prevention and treatment. Currently, drugs targeting these pathways, such as aspirin, S1PR modulators and PKCδ inhibitors [61, 70–73], are already in clinical use or under investigation [74]. Moreover, combination therapies potentially offer greater efficacy due to the interconnected nature of lipid signalling.

Our study further emphasizes the need for personalized treatment strategies tailored to the disease stage and pathophysiological characteristics of patients. Systemic immunosuppression, a well-documented feature of the subacute phase, is characterized by lymphopenia and increased monocyte differentiation [53, 75, 76], consistent with our data. The broad correlation between lipid pathways and inflammatory markers highlights the role of membrane lipids in IS immunosuppression. However, post-stroke inflammation varies by phase: acute phase is marked by acute inflammation, subacute phase by immune suppression, and chronic phase by chronic low-grade inflammation [77]. Consequently, inflammation-targeted therapies need dynamic monitoring of lipid mediators and cytokines for precise inflammatory status assessment and personalized treatment.

Sphingolipids, particularly S1P, show potential prognostic value, supported by the inverse correlations of sphingolipids (Cer-VLPUFAs, S1P, *SPHK2* and *S1PR1*) with NIHSS scores. The role of very-long-chain Cers in neuronal development and apoptosis suppression has been preliminarily validated [78, 79], suggesting the accumulation of Cer-VLPUFA may contribute to improved clinical outcomes. Low serum S1P levels are associated with great disease severity, large infarct volumes, and poor outcomes [80, 81], likely due to the neuroprotective role of the SPHK2-S1P-S1PRs signalling. SPHK2-dependent S1P production reduces lesion size and improves neurological function [82–85]. S1PR1 and S1PR5 contribute to endothelial function maintenance, neurogenesis, and myelination [86–90]. S1PR modulator fingolimod have shown therapeutic benefits in phase II clinical trials, including reduced neurological deficits and improved recovery in IS [61, 71–73], highlighting S1P-S1PRs signalling’s therapeutic promise. Future research should further investigate the dual potential of S1P-S1PRs signalling as both therapeutic targets and prognostic biomarkers.

Study limitations include mismatched demographics, despite adjustments. Future studies should use larger, matched cohorts for validation. Erythrocyte may not fully represent lipid dynamics in other tissues. Cross-sectional design restricts causality; prospective studies and longitudinal animal studies are essential to explore membrane lipid changes before and after IS.

## Conclusions

Our study demonstrates that impaired erythrocyte membrane lipid homeostasis, particularly in the GPL-LPL, PLC-IP3/DG, and S1P-S1PRs signalling pathways, plays a pivotal role in IS pathology by regulating thrombosis and inflammation. These findings highlight the therapeutic potential of targeting these lipid pathways and the prognostic value of S1P signalling. Future research should focus on elucidating the dynamic mechanisms of lipid-mediated regulation and exploring the interplay between these pathways to develop more effective and personalized therapeutic strategies for IS.

## Electronic supplementary material

Below is the link to the electronic supplementary material.

Supplementary Material 1

Supplementary Material 2

Supplementary Material 3

## Data Availability

The datasets used and/or analysed during the current study are available from the corresponding author on reasonable request.

## Abbreviations

AA: Arachidonic acid
ACSL4: Acyl-CoA synthetase long-chain family member 4
BMI: Body mass index
CDase: Ceramidase
CerS: Ceramide synthase
CI: Coagulation index
CV: Coefficient of variation
DG: Diglyceride
DGK: Diacylglycerol kinase
ELISA: Enzyme-linked immunosorbent assay
FDR: False discovery rate
GL: Glycerolipid
GPL: Glycerophospholipids
HC: Healthy control
HexCer: Hexosylceramide
IL-1β: Interleukin-1β
IL-6: Interleukin-6
IL-10: Interleukin-10
IP3: Inositol 1,4,5-trisphosphate
IP3R: Inositol 1,4,5-trisphosphate receptor
IS: Ischemic stroke
K: Clotting time
LDL-C: Low-density lipoprotein cholesterol
LPC: Lysophosphatidylcholine
LPE: Lysophosphatidylethanolamine
LPI: Lysophosphatidylinositol
LPL: Lysophospholipid
LPS: Lysophosphatidylserine
Lym: Lymphocyte
MA: Maximum amplitude
Mono: Monocyte
MPAR: The maximum platelet aggregation rate
MUFA: Monounsaturated fatty acid
Neu: Neutrophil
NIHSS: National Institutes of Health Stroke Scale
OPLS-DA: Orthogonal partial least squares-discriminant analysis
ox-LDL: Oxidized low-density lipoprotein
PA: Phosphatidic acid
PC: Phosphatidylcholine
PCA: Principal component analysis
PE: Phosphatidylethanolamine
PG: Phosphatidylglycerol
PI: Phosphatidylinositol
PIP2: Phosphatidylinositol-4,5-phosphate
PKC: Protein kinase C
PLA2: Phospholipase A2
PLC: Phospholipase C
PPI: Phosphoinositide
PS: Phosphatidylserine
PUFA: Polyunsaturated fatty acid
QC: Quality control
QE-MS: Q Exactive plus Mass spectrometer
R: Reaction time
Rt: Retention time
S1P: Sphingosine-1-phosphate
S1PR: Sphingosine-1-phosphate receptor
SFA: Saturated fatty acid
SM: Sphingomyelin
SPHK: Sphingosine kinase
TEG: Thromboelastogram
TG: Triglyceride
VLPUFA: Very long-chain polyunsaturated fatty acid
WBC: White blood cell count
WGCNA: Weighted gene co-expression network analysis
α: Alpha angle
