# Supplementary material for: Impaired membrane lipids in ischemic stroke: a key player in inflammation and thrombosis
Source: J Neuroinflammation. 2025 May 28;22:144. doi: 10.1186/s12974-025-03464-w (PMC12117946; doi:10.1186/s12974-025-03464-w)
Supplement: Supplementary file 1 — Supplementary Material 1 [file 12974_2025_3464_MOESM1_ESM.pdf]

Supplementary Materials

Impaired membrane lipids in ischemic stroke: a key player in inflammation and thrombosis

Qian Wang, Dandan Wang, Yan Gao, Jie Jiang, Minghui Li, Shuhui Li, Xiaowen Hu, Jinfeng Wang, Tianqi Wang, Juan Zhang, Lei Feng, Chao Quan, Ping Zhang, Lan Zheng, Chunling Wan

Supplementary tables

Supplementary table 1. Curves information for six standard lipids

Standard	Quantified lipid classes	Ion model	Standard curve	Applicable peak area range	R^2
SM (d18:1/12:0)	SM, LSM	positive	y = 0.0000000083 x - 0.0007287390	x < 7.94E+06	0.9998
			y = 0.0000000053 x + 0.1587767613	x ≥7.94E+06	0.9991
		negtive	y = 0.0000000668 x + 0.0040458814	x < 1.17E+07	0.9998
			y = 0.0000000712 x - 0.5681458688	x ≥1.17E+07	0.9999
Cer(d18:1/18:1)	Cer, CerP, HexCer	positive	y = 0.0000000066 x + 0.0000856560	x < 2.96E+07	0.9996
			y = 0.0000000076 x - 0.1413284372	x ≥2.96E+07	0.9993
		negtive	y = 0.0000000084 x - 0.0003113588	x < 2.34E+07	0.9997
			y = 0.0000000109 x - 0.2932891407	x ≥2.34E+07	0.9972
PS (17:0/17:0)	PS, LPS, PI, LPI	positive	y = 0.0000000770 x + 0.0636385768	x < 3.36E+07	0.9997
			y = 0.0000000435 x + 1.2389978926	x ≥3.36E+07	0.9979
		negtive	y = 0.0000000738 x - 0.0350880269	x < 9.45E+06	0.9989
			y = 0.0000000616 x + 0.6747682138	x ≥ 9.45E+06	0.9991
PE (17:0/17:0)	PE, PA, PG, LPE,	positive	y = 0.0000000281 x - 0.0051412177	x < 1.30E+07	0.9992
			y = 0.0000000226 x + 0.2128597948	x ≥1.30E+07	0.9997
		negtive	y = 0.0000000349 x - 0.0041971398	x < 9.65E+06	0.9998
			y = 0.0000000372 x - 0.2243384129	x ≥9.65E+06	0.9994
PC (19:0/19:0)	PC, LPC	positive	y = 0.0000000469 x + 0.0005795119	x < 3.46E+06	0.9999
			y = 0.0000000459 x - 0.2577163938	x ≥3.46E+06	0.9815
		negtive	y = 0.0000000270 x - 0.0076944786	x < 6.31E+06	0.9999
			y = 0.0000000344 x - 0.8500114095	x ≥ 6.31E+06	0.9925
TG (17:0/17:1/17:0)D5	TG, DG	positive	y = 0.0000000042 x + 0.0005758807	x < 3.68E+07	0.9998
			y = 0.0000000024 x + 0.1168969206	x ≥3.68E+07	0.9998

The standard curve equation was derived by fitting the data points of standard concentration gradients through linear regression analysis, where x represents the peak area and y denotes the concentration in units of μg/mL. SM: Sphingomyelin; LSM: Lysosphingomyelin; Cer: Ceramide; CerP: Ceramide phosphate; HexCer: Hexosyl ceramide; PS: Phosphatidylserine; LPS: Lysophosphatidylserine; PI: Phosphatidylinositol; LPI: Lysophosphatidylinositol; PE: Phosphatidylethanolamine; PA: Phosphatidic acid; PG: Phosphatidylglycerol; LPE: Lysophosphatidylethanolamine; PC: Phosphatidylcholine; LPC: Lysophosphatidylcholine; TG: Triglyceride; DG: Diglyceride.

Supplementary table 2. Primer sequences of lipid signalling pathways related genes

Gene	Protein name	Forward（5'-3'）	Reverse（3'-5'）
PLA2G4A	Cytosolic Phospholipase A2	TGAAGTTTGCTCATGCCCAG	CACATCACGTGCAGAATGCAA
PLA2G6	Calcium-Independent Phospholipase A2	CCTGGATGGAGGAGGAGTGA	AGGCCATGGACTTACTGTGC
ACSL4	Long-Chain Fatty-Acid-Coenzyme A Ligase 4	AGGACATTTAAAAACGCTATGGCA	GTCCCAAGGCTGTCCTTCTT
PLCB1	Phospholipase C Beta 1	GGTGCAGTATATCAAGAGGCTAGAA	CACCTGCAGCTTGGGCTTTT
PLCB2	Phospholipase C Beta 2	GAACCATGTGGACTCACCCC	CACACTGTGCCCTCACCTG
PLCB3	Phospholipase C Beta 3	CCGGCCTGATGAGTTTTCCT	GTCAGGTATGGCTTGCCCTT
PLCG1	Phospholipase C Gamma 1	TTCTGCGCTTCGTGGTGTAT	ACCATTCTCCTGCTTGGCA
PLCG2	Phospholipase C Gamma 2	GCACTCAATTTCCAGACGGC	GAGCACCGAGAACCTTGACT
ITPR1	Inositol 1,4,5-Trisphosphate Receptor Type 1	GAGTTTCAGCCCTCAGTGGA	GCAGAGTGGTGGGATCTAGC
ITPR2	Inositol 1,4,5-Trisphosphate Receptor Type 2	TCAGCACCTTGGGGTTAGTG	TGTGGTTCCCTTGTTTGGCT
ITPR3	Inositol 1,4,5-Trisphosphate Receptor Type 3	GTGTGACGAGTACAAGGGCA	AAGCGGTACAAGCCATTCCA
PPKCB	Protein Kinase C Beta	CCATCTGCAAAGGGCTGATGA	TCTCTTGTCTCTAGCTTTTGGCT
PPKCD	Protein Kinase C Delta	GTTGGTGCGTTGTAGCAGC	TAGGAGTTGAAGGCGATGCG
PPKCE	Protein Kinase C Epsilon	AGCTGGCTGTCTTTCACGAT	TGTCTTTAGGGGCTTCACCC
PLD1	Phospholipase D1	TACCGGGTCCATCCGTAGTT	GGCGTGGAGTACCTGTCAAT
PLD2	Phospholipase D2	TACCAGCGGATCCAAGGTGG	CTGGAGAATAGGCAACGGCA
DGKQ	Diacylglycerol Kinase Theta	GTTCTACGTGGCAGAGAGCA	AGGCAACGTCCAACACTACC
DGKG	Diacylglycerol Kinase Gamma	GTGCCGGATGACGTTTCAC	GACTTCTCACCTGGCCTGTC
ASAH1	N-Acylsphingosine Amidohydrolase 1	GTGGCGTTGGCTGCTAGA	AACTGCACCTCTGTACGTTG
ACER3	Alkaline Ceramidase 3	GCACCTACCATAGACCTGGC	TGGGTGGCAGCATTAGTCAG
SPHK1	Sphingosine Kinase 1	ATGCTGGCTATGAGCAGGTC	ACATCAGCAATGAAGCCCCA
SPHK2	Sphingosine Kinase 2	ACCTACGAAGAGAACCGTGC	ACCAATAGAAGCAACCGGGG
S1PR1	Sphingosine-1-Phosphate Receptor 1	GCTCTCCGAACGCAACTTCG	AGGGGTGGTTCGATGAGTGA
S1PR4	Sphingosine-1-Phosphate Receptor 4	AAGTTGCAGTCTTGCGTGTG	GTTCCCTGCTCCCCATACAG
S1PR5	Sphingosine-1-Phosphate Receptor 5	AGTCGGAAAAGATGCAGGGG	GTGTTCCCAAGCAGAACGTC
CERS2	Ceramide Synthase 2	ACTCCCTCTTGATGCCCTCC	CTGAGGCTTTGGCGTAGACA
CERS4	Ceramide Synthase 4	TCTGCAGCTAAAGAACGGGG	GCTATGTGGCTGTTGTGTGC
ACTB	Actin Beta	ACAGAGCCTCGCCTTTG	CCTTGCACATGCCGGAG
GAPDH	Glyceraldehyde-3-Phosphate Dehydrogenase	AAAATCAAGTGGGGCGATGC	TGGTTCACACCCATGACGAA

Supplementary table 3. Demographic and clinical characteristics of patients with ischemic stroke and healthy control

Items	Ischemic stroke (n = 56)	Healthy control (n = 55)	*p*-value
Gender (male/female)	36/19 (70.6%)	15/41 (27.3%)	< 0.0001[A]
Age (years, Mean ±SEM),	58.46 ± 1.10	49.48 ± 0.97	< 0.0001[B]
BMI (kg/m2, Mean ±SEM)	25.14 ± 0.44	23.41 ± 0.41	0.005[B]
Hypertension	40 (71.4%)	0/55 (0%)	< 0.0001[A]
Diabetes	24 (42.9%)	0/55 (0%)	< 0.0001[A]
Hyperlipidaemia	5 (8.9%)	0/55 (0%)	0.057[A]
Triglyceride (mmol/L, Mean ±SEM)	1.90 ± 0.13	1.82 ± 0.32	0.002[B]
High-density lipoprotein cholesterol (nmol/L, Mean ±SEM)	1.00 ± 0.03	1.41 ± 0.05	< 0.0001[B]
Low density lipoprotein cholesterol (mmol/L, Mean ±SEM)	2.87 ± 0.12	3.20±0.12	0.132[B]
Glucose (mmol/L, Mean ±SEM)	7.18 ± 0.35	5.16 ± 0.08	< 0.0001[B]

[A]. *p*-values were calculated by the chi-square test.

[B]. *p*-values were calculated by the Mann-Whitney *U* test.

BMI: Body Mass Index.

Supplementary figures

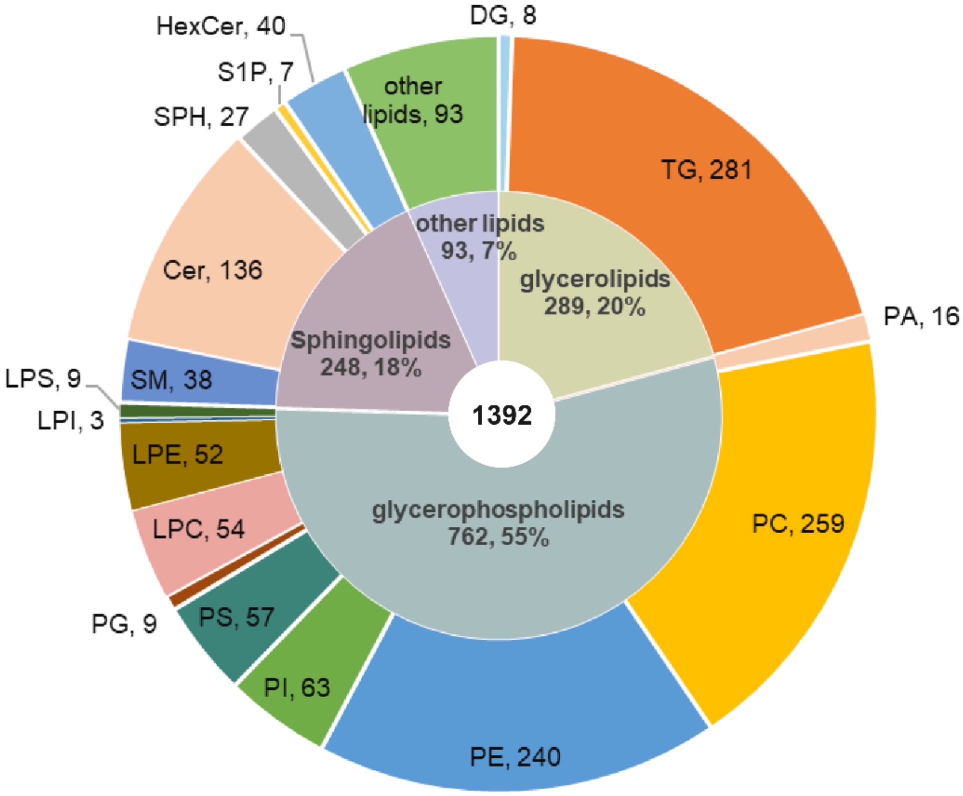

Supplementary Figure 1. The primary lipid classes in the erythrocyte membrane identified qualitatively based on the LipidSearch database. The numbers represent the quantity of each lipid class, while the percentages (%) indicate the proportion of the number of lipids in different classes to the total lipids.

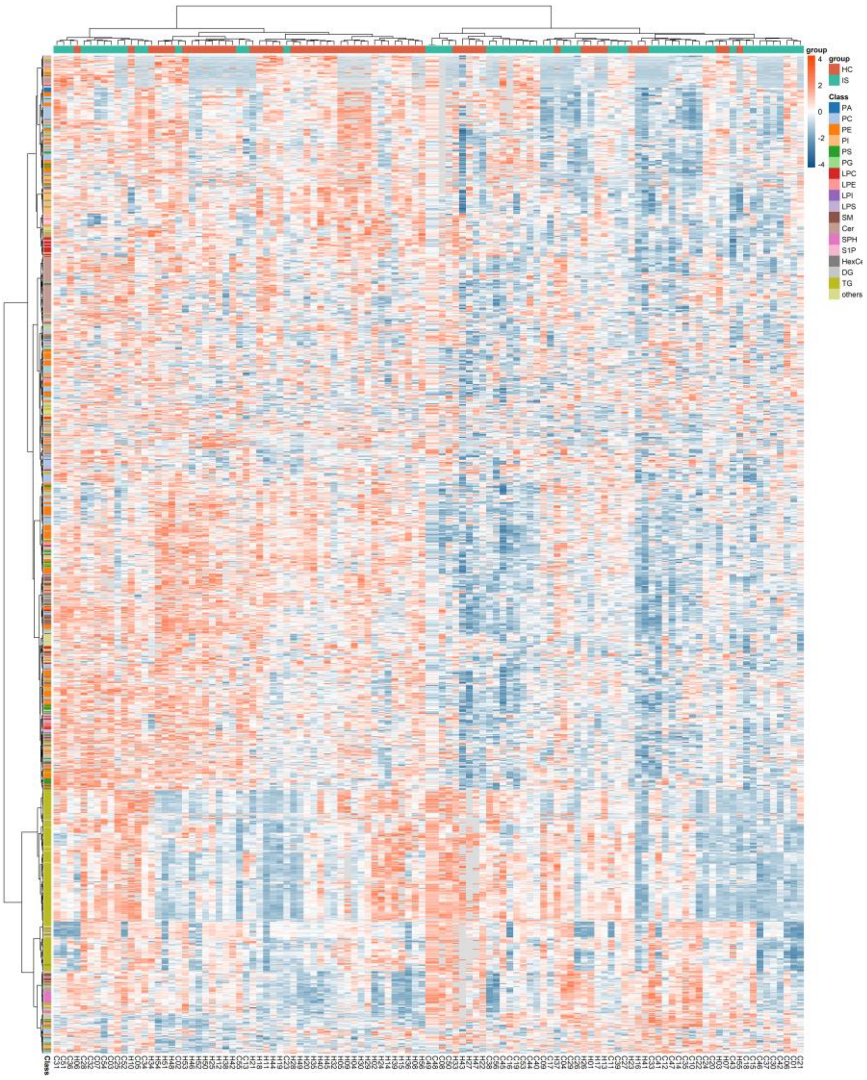

Supplementary Figure 2. Unsupervised cluster analysis of 1392 lipids detected in ischemic stroke (IS) and healthy controls (HC) groups.

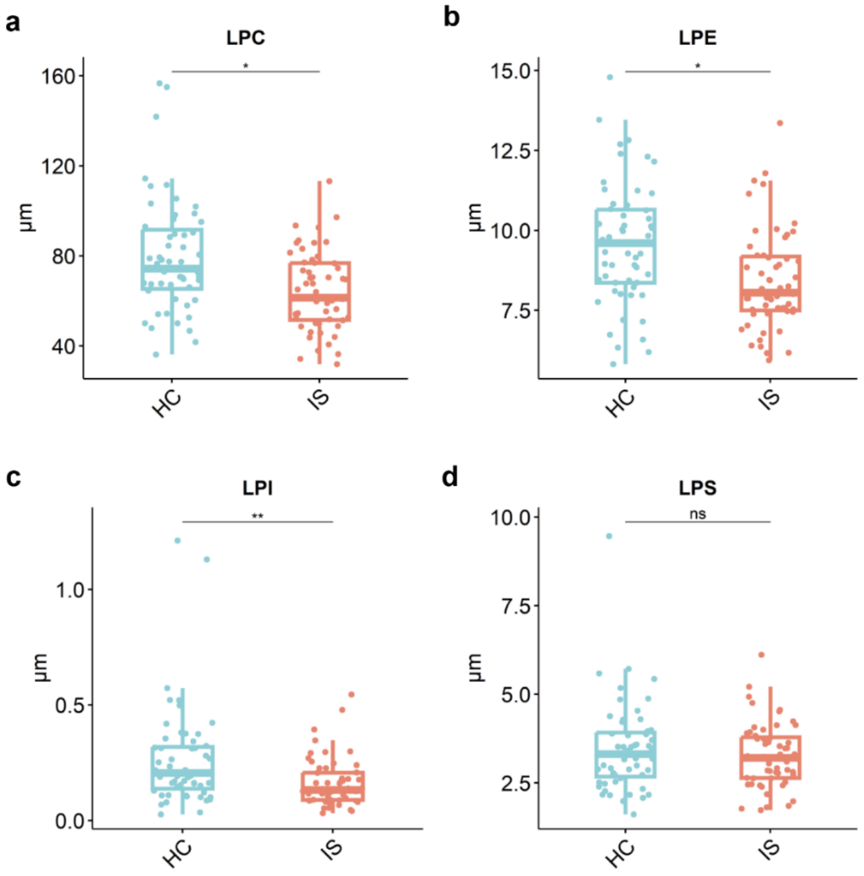

Supplementary Figure 3. The total content of LPC, LPE, LPI, and LPS in the healthy controls (HC) and ischemic stroke (IS) groups. All p-values were calculated by multiple linear regression adjusting for gender, age, and Body Mass Index. $*p < 0.05$, $**p < 0.01$.

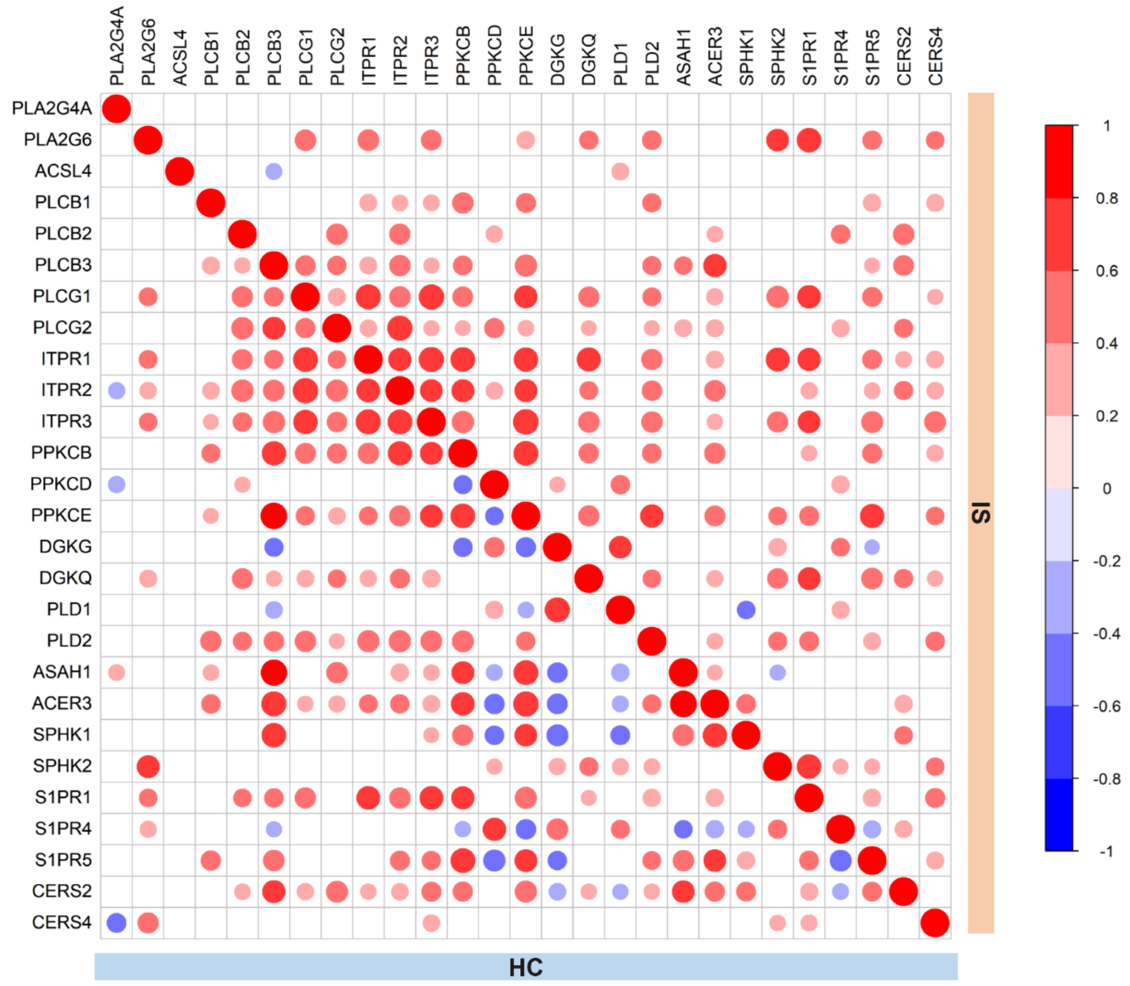

Supplementary Figure 4. Correlation analysis of genes involved in the disturbed lipid signalling pathways in the healthy controls (HC) (bottom) and ischemic stroke (IS) (top). The correlation coefficients and *p*-values were calculated by Spearman Rank Correlation Test. All displayed dots were statistically significant ($p < 0.05$).

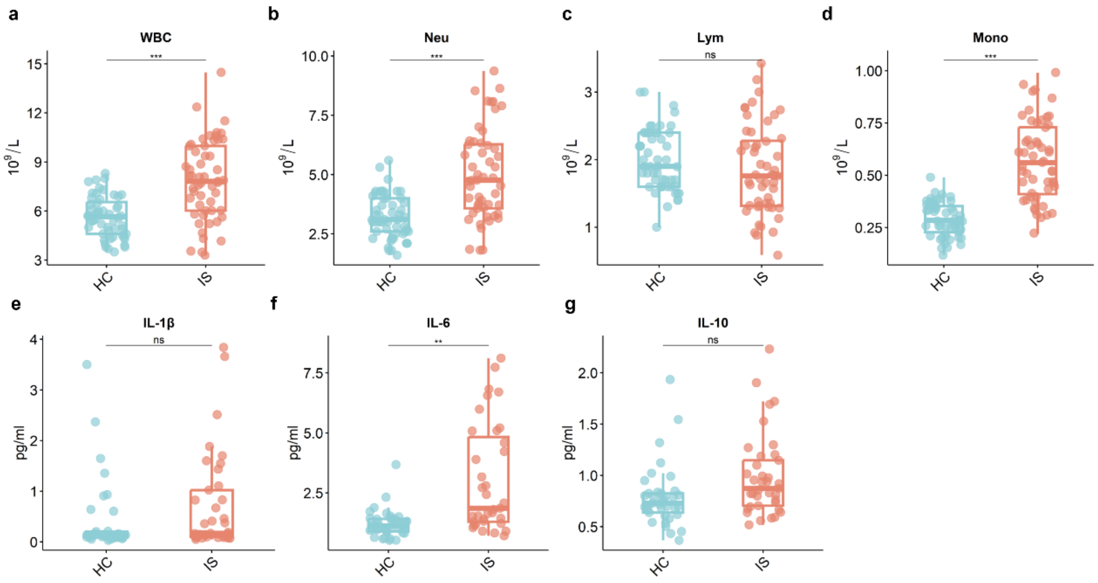

Supplementary Figure 5. Differences in inflammatory markers between the healthy controls (HC) and ischemic stroke (IS) groups. WBC: White blood cell count; Neu: Neutrophils; Lym: Lymphocytes; Mono: Monocytes; IL-1β: Interleukin-1β; IL-6: Interleukin-6; IL-10: Interleukin-10. All *p*-values were calculated by multiple linear regression adjusting for gender, age, and Body Mass Index. $^{**}p < 0.01$, $^{***}p < 0.001$.

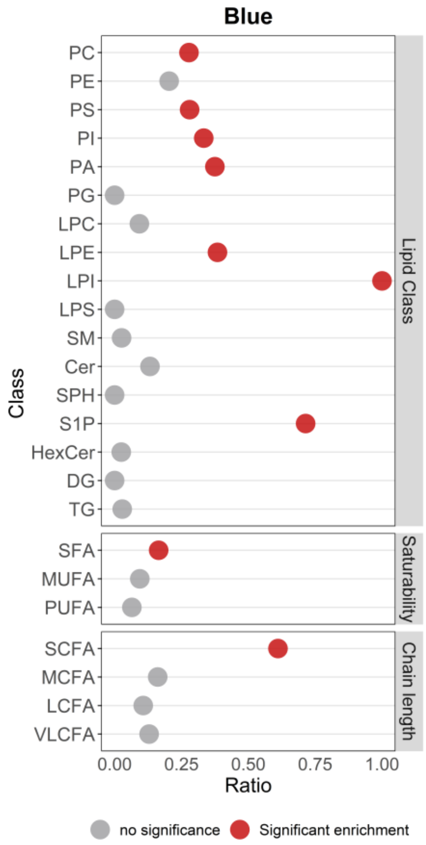

Supplementary Figure 6. The result of lipid enrichment analysis for the blue module. The ratio represents the proportion of a specific lipid classification within the module relative to the total lipid quantity. *p*-values were calculated by hypergeometric test, with $p < 0.05$ regarded as significantly enriched.
