# Supplementary material for: Impaired membrane lipids in ischemic stroke: a key player in inflammation and thrombosis
Source: J Neuroinflammation. 2025 May 28;22:144. doi: 10.1186/s12974-025-03464-w (PMC12117946; doi:10.1186/s12974-025-03464-w)
Supplement: Supplementary file 3 — Supplementary Material 3 [file 12974_2025_3464_MOESM3_ESM.pdf]

Figure Legends

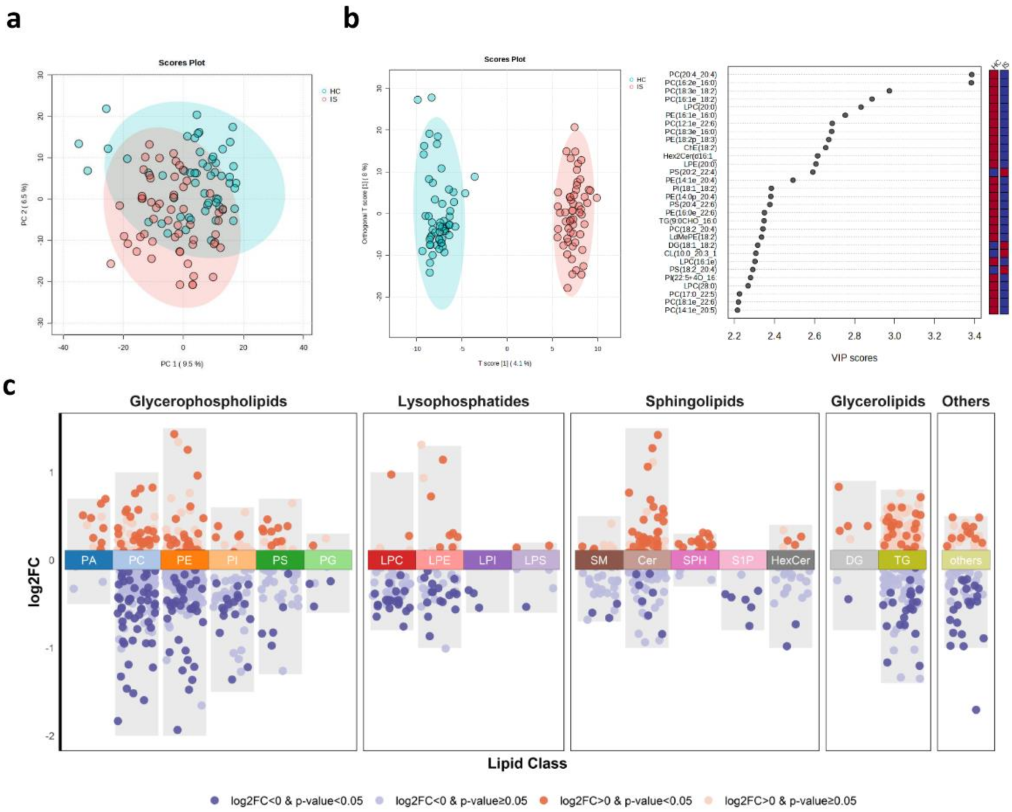

Figure 1. Impaired erythrocyte membrane lipid homeostasis in patients with ischemic stroke (IS). (a) 2D score plot of principal component analysis of IS and HC groups. (b) 2D score plot of orthogonal partial least squares discriminant analysis (OPLS-DA) of IS and HC groups (left); and the top 30 lipids with the highest variable importance in projection (VIP) scores given by OPLS-DA (right). (c) Differences in 1392 lipids between IS and HC groups. *p*-values were calculated by multiple linear regression adjusting for gender, age, and Body Mass Index. FC: Fold change; HC: Healthy controls.

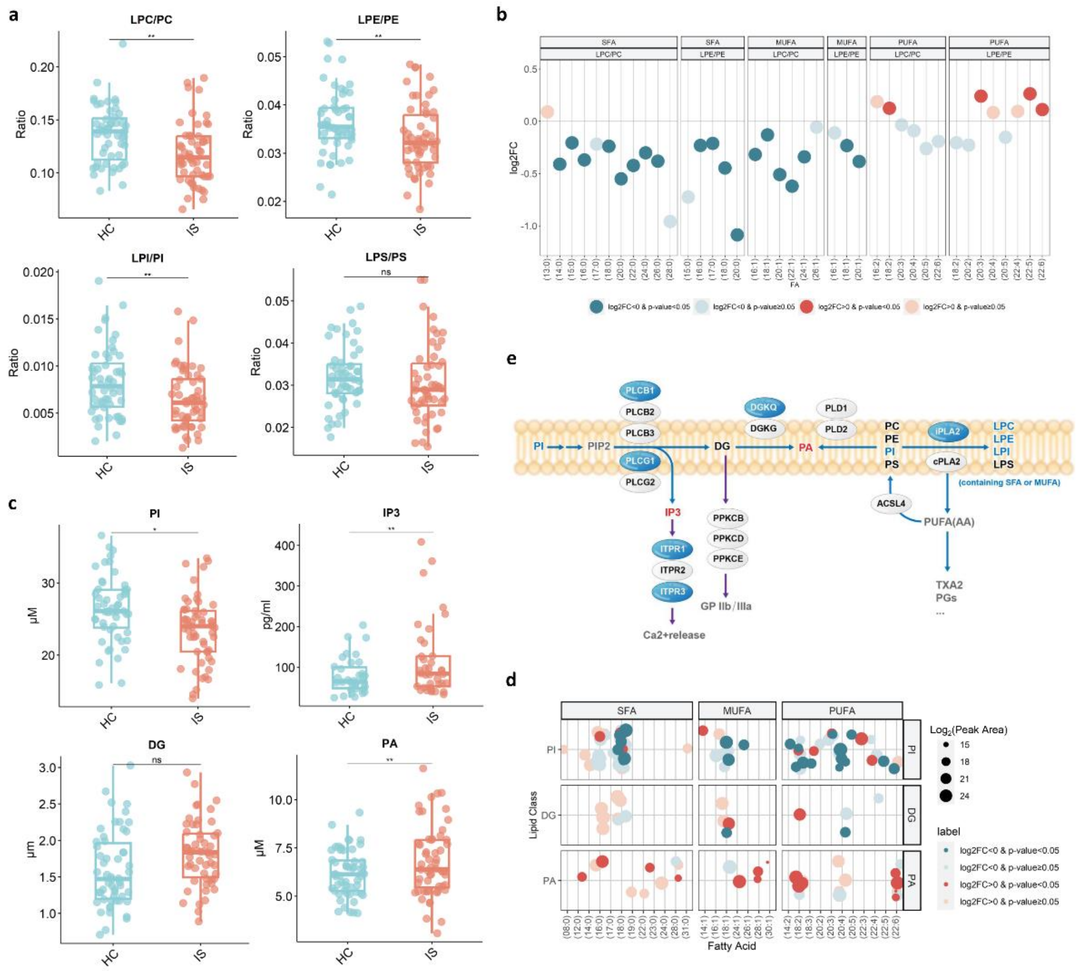

Figure 2. Disturbed glycerophospholipid (GPL) metabolism in patients with ischemic stroke (IS). (a) Ratios of LPLs to GPLs in patients with IS and healthy controls (HC), including LPC/PC, LPE/PE, LPI/PI, and LPS/PS. (b) Differences in the ratio of the total content of GPLs containing specific fatty acyl chains to the total content of LPLs containing specific fatty acyl chains between the HC and IS groups. (c) The total content of PI, IP3, DG, and PA in the HC and IS groups. (d) Differences of individual lipids of PI, PA and DG classes in patients with IS and HC. Each GPL contains two fatty acyl chains of different chain lengths and saturations. The horizontal coordinates indicate the individual fatty acyl chains contained in PIs, DGs, and PAs, so each lipid corresponds to two dots. Dot color represents significance and log_2FC

positive or negative, and dots size represents the $\log_2$(Peak Area). (e) Schematic diagram of changes in lipid contents and genes expression associated with GPL signalling pathways in the IS group compared to the HC group. Non-italics indicate lipids, and italics indicate genes. For lipids, blue letters indicate a significant decrease in the IS group, red letters indicate a significant increase in the IS group, and black letters represent no significant change. For genes, blue ovals represent significantly downregulated, and white ovals represent no significant change. Blue lines represent lipid synthesis or hydrolysis, and the purple lines represent regulatory targets or receptors. All *p*-values were calculated by multiple linear regression adjusting for gender, age, and Body Mass Index. $*p < 0.05$, $**p < 0.01$, $***p < 0.001$. LPL: lysophosphatide; FC: Fold change; SFA: Saturated fatty acids; MUFA: Monounsaturated fatty acids; PUFA: Polyunsaturated fatty acids.

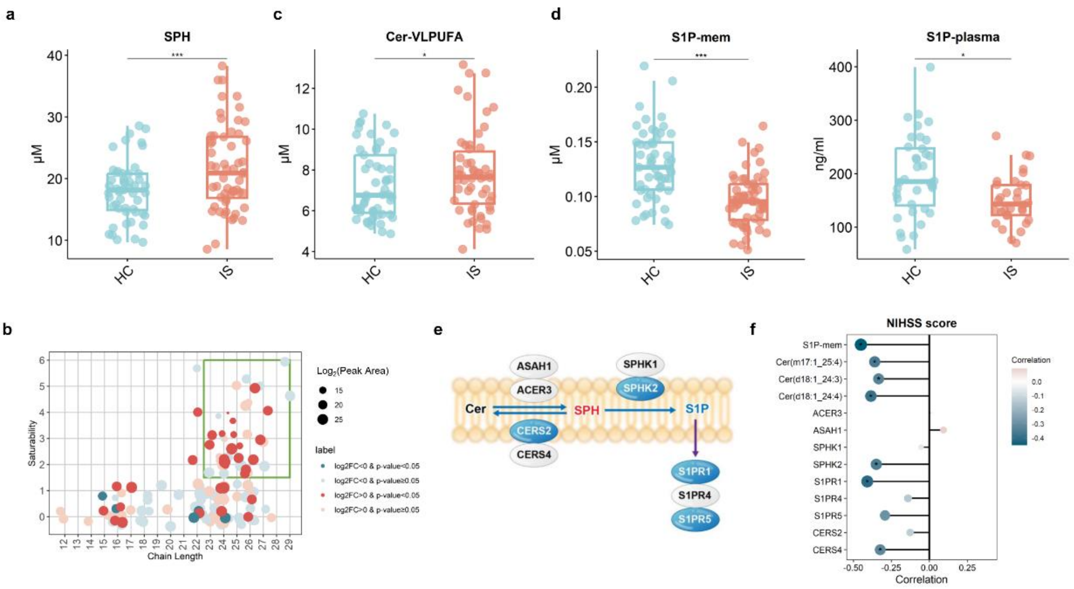

Figure 3. Disturbed sphingolipid (SP) metabolism in patients with ischemic stroke

(IS). (a) The total content of sphingosine (SPH) in the healthy controls (HC) and IS groups. (b) Differences of individual lipids in ceramide (Cer) class between HC and IS groups. Each dot represents an individual lipid. Cer comprises a SPH backbone (usually chain length C18) attached to a variable chain length fatty acid (FA) via amide bon, and the horizontal and vertical coordinates indicate the chain length and saturability of the fatty acyl chains, respectively. Dot color represents significance and $\log_2$FC positive or negative, and dots size represents the $\log_2$ (Peak Area). Dots in the green box are Cer-VLPUFAs. (c) The total content of Cer-VLPUFA in the HC and IS groups. (d) The total content of erythrocyte membrane sphingosine-1-phosphate (S1P) (left) and plasma S1P (right) in the HC and IS groups. (e) Schematic diagram of changes in lipid contents and genes expression related to sphingolipid metabolism in the IS group compared to the HC group. Non-italics indicate lipids, and italics indicate genes. For lipids, blue letters indicate a significant decrease in the IS group, red letters indicate a significant increase in the IS group, and black letters represent no significant change. For genes, blue ovals represent significantly downregulated, and white ovals represent no significant change. Blue lines represent lipid synthesis or hydrolysis, and the purple lines represent regulatory targets or receptors. (f) NIHSS score was significantly correlated with sphingolipids and related genes in IS. *p*-values were calculated by multiple linear regression adjusting for gender, age, and Body Mass Index (a-d) or Spearman rank correlation Test (f) as appropriate. $^*p < 0.05$, $^{***}p < 0.001$. FC: Fold change; VLPUFA: Very long chain polyunsaturated fatty acids; NIHSS: National institutes of health stroke scale.

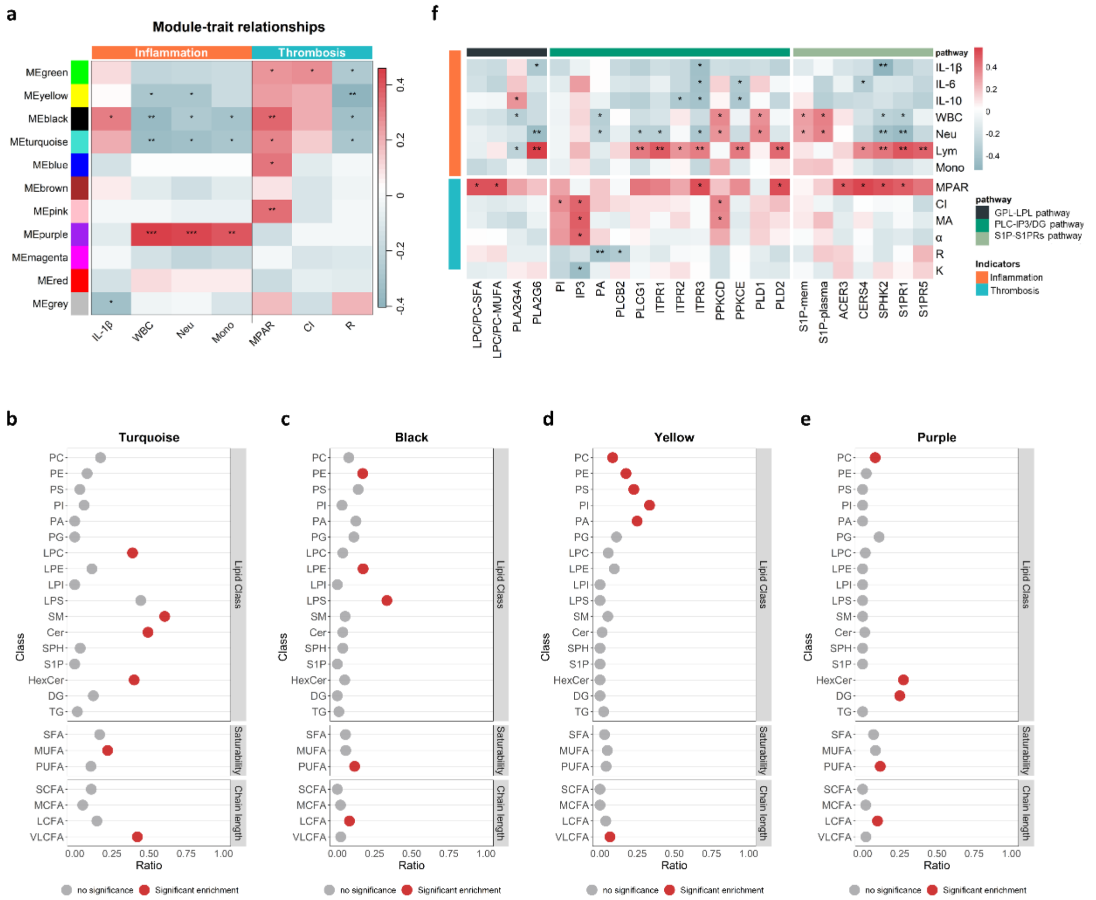

Figure 4. Disturbed lipid metabolism pathways are synergistically involved in inflammation and thrombosis in IS. (a) 11 modules revealed by the WGCNA. (b-e) The results of lipid enrichment analysis for the turquoise (b), black (c), yellow (d), and purple (e) modules. The ratio represents the proportion of a specific lipid classification within the module relative to the total lipid quantity. p-values were calculated by hypergeometric test, with $p < 0.05$ regarded as significantly enriched. (f) Spearman Rank correlation analysis of clinical markers of inflammation and thrombosis with genes and lipids in lipid signalling pathways in IS. *$p < 0.05$, **$p < 0.01$. IS: Ischemic stroke; NIHSS: National institutes of health stroke scale; WGCNA, Weighted gene co-expression network analysis; SFA: Saturated fatty acids; MUFA: Monounsaturated

fatty acids; PUFA: Polyunsaturated fatty acids; SCFA: Short chain fatty acid; MCFA: Medium chain fatty acids; LCFA: Long chain fatty acid; VLCFA: Very long chain fatty acids; IL-6: Interleukin-6; IL-1β: Interleukin-1β; IL-10: Interleukin-10; WBC: White blood cell count; Neu: Neutrophils; Lym: Lymphocytes; Mono: Monocytes; MPAR: The maximum platelet aggregation rate; The five indices in the thromboelastogram, including CI, MA, α, R, and K. CI: Coagulation index, represents a composite score that reflects the overall coagulation status of the patient; MA: Maximum amplitude, measures the maximum strength of the clot; α: Alpha angle, represents the rate of clot formation; R: Reaction time, is the time taken for the initial clot formation to begin; K: Clotting time, measures the time taken for the clot to reach a certain amplitude (typically 20 mm).

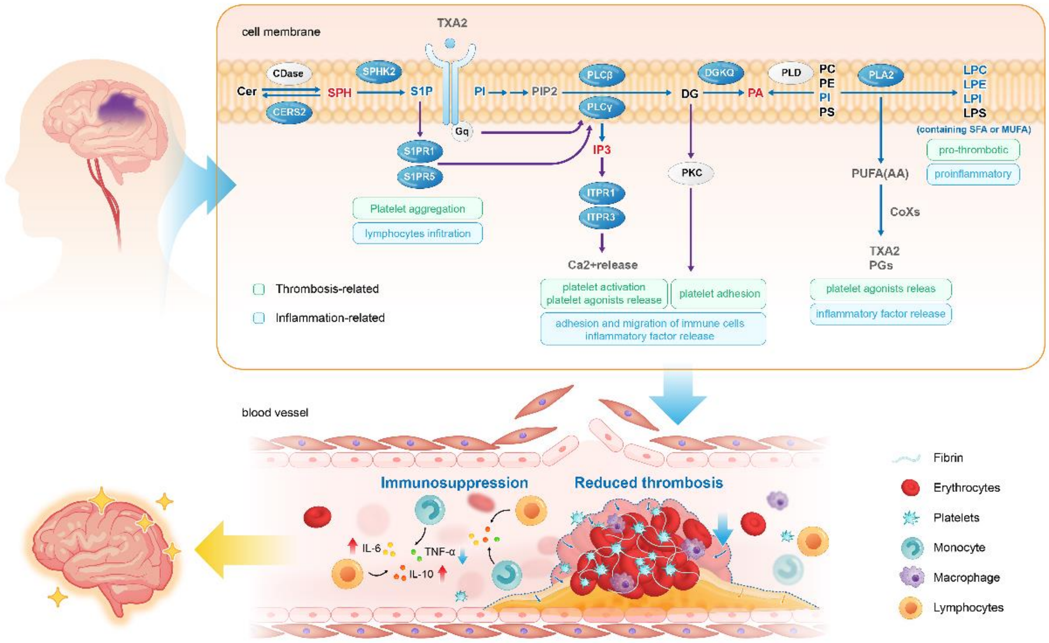

Figure 5. Pathological mechanisms associated with membrane lipid homeostasis disorder in ischemic stroke (IS). Membrane lipid homeostasis is impaired in patients

with IS. The impaired lipids are primarily concentrated in three disturbed lipid signalling pathways: GPL-LPL, IP3/DG, and S1P-S1PRs pathway. These lipid pathways interact to form a highly interconnected lipid signalling network that collectively participates in thrombosis and the inflammatory responses of IS. However, during the subacute phase following IS, the key gene expressions and lipid levels significantly decrease, including a decline in the expression of PLA2, PLCs, IP3Rs, SPHK2, and S1PRs, as well as a reduction in the levels of PI, LPLs containing SFA or MUFA, and S1P. This indicates that these pathways are suppressed during this phase, thereby diminishing their roles in mediating inflammatory responses and thrombosis, which ultimately reduces pathological damage and accelerates homeostasis restoration. PLA2: Phospholipase A2; GPL: Glycerophospholipid; LPL: Lysophospholipid; PLC: Phospholipase C; IP3: Inositol 1,4,5-trisphosphate; PI: Phosphatidylinositol; DG: Diglyceride; SPHK2: Sphingosine kinase 2; S1P: Sphingosine-1-phosphate; S1PR: S1P receptor; IP3R: IP3 receptor; SFA: Saturated fatty acid; MUFA: Monounsaturated fatty acid.
